# The *Brachiacantha* Dejean, 1837 (Coleoptera, Coccinellidae) of Central America

**DOI:** 10.3897/zookeys.1024.56927

**Published:** 2021-03-16

**Authors:** Jorge Ismael Nestor-Arriola, Víctor Hugo Toledo-Hernández, Ángel Solís, Guillermo González, Jaroslav Větrovec

**Affiliations:** 1 Centro de Investigación en Biodiversidad y Conservación (CIByC), Universidad Autónoma del Estado de Morelos. Av. Universidad #1001, Col. Chamilpa, Cuernavaca, Morelos, C. P. 62209, México Universidad Autónoma del Estado de Morelos Cuernavaca Mexico; 2 Departamento de Historia Natural, Museo Nacional de Costa Rica. Santo Domingo de Heredia, Costa Rica Museo Nacional de Costa Rica Santo Domingo de Heredia Costa Rica; 3 La Reina, Santiago, Chile Unaffiliated Santiago Chile; 4 Buzulucka, Hradec Kralove, Česká Republika Unaffiliated Buzulucka Czech Republic

**Keywords:** Cusps, holotypes, Hyperaspidini, key, synonyms, tooth

## Abstract

A revision of the Central American species of the genus *Brachiacantha* was undertaken to update the knowledge of the Central American species of the genus. Material of several collections was reviewed, using original descriptions and keys, and comparing with the type material. Twenty-five species of the genus *Brachiacantha* were found in Central American material, including nine new species: *B. nubes* Nestor-Arriola, Toledo-Hernández and Solís, **sp. nov.**, *B. dentata* Nestor-Arriola, Toledo-Hernández and Solís, **sp. nov.**, *B. isthmena* Nestor-Arriola, Toledo-Hernández and Solís, **sp. nov.**, *B. aurantiapleura* Nestor-Arriola, Solís and Toledo-Hernández, **sp. nov.**, *B. invertita* Nestor-Arriola, Toledo-Hernández and Solís, **sp. nov.**, *B. papiliona* Nestor-Arriola, Toledo-Hernández and Solís, **sp. nov.**, *B. tica* Nestor-Arriola, Toledo-Hernández and Solís, **sp. nov.**, *B. hexaspina* González, Větrovec and Nestor-Arriola, **sp. nov.**, and *B. mimica* Nestor-Arriola and Toledo-Hernández, **sp. nov.** Nomenclatural changes include *Brachiacantha gorhami* (Weise), **comb. nov.**, *B. guatemalensis* (Gorham), **comb. nov.**, and *Brachiacantha duodecimguttata* Leng, **syn. nov**. for *B. lepida* Mulsant. The male genitalia of the species *B. fenestrata* Gorhan, *B. octostigma* Mulsant, *B. aperta* Weise, and *B. cachensis* Gorhan are described and illustrated for the first time. New records include *B. indubitabilis* Crotch and *B. bipartita* Mulsant (Costa Rica and Guatemala), *B. gorhami* (Weise) (El Salvador), and *B. cachensis* Gorham (Panamá). A key to the species is included.

## Introduction

The genus *Brachiacantha* Dejean, 1837, belongs to the family Coccinellidae, also known as ladybugs, lady beetles, ladybirds or ladybird beetles. The most evident character of the genus is the tooth on the leading edge of the protibia; however, this tooth is variable in size and is very small in some species (Gordon 1985; Gordon et al. 2014). Twelve species were listed for Central America, México, and the United States in the Biologia Centrali-Americana (Gorham 1894). Some species were added later by Weise (1903) and Leng (1911). Several of the above-mentioned studies do not include a description of the male genitalia of species, which is one of the structures most commonly used to recognize species today. The genus belongs to the tribe Brachiacanthini, but Seago et al. (2011) synonymized this tribe with Hyperaspidini.

Leng (1911) was the first to divide the genus into groups; he described six groups based on external characters; however, some of the groups were inaccurate (Nestor-Arriola and Toledo-Hernández 2016). Gordon (1985) revised the North American species and divided them into four groups based on external characters and male genitalia; some of his groups resemble those of Leng (1911). Gordon et al. (2014) revised the species of South America and divided them into twelve groups based on male genitalia, and in only a few groups on external characters and female genitalia. Some of the South American groups are equated with those of North America; the *bistripustulata* group (Gordon et al. 2014) have the same diagnostic characters of the *dentipes* group (Gordon 1985). The same applies to the North American *ursina* group and the South American *debbie* group; therefore, the known *Brachiacantha* species can be separated into fourteen valid groups.

## Materials and methods

The material of the *Brachiacantha* species studied comes from several collections. The acronyms used in the text are as follows:

**CEAM** Colección Entomológica del Colegio de Posgraduados, Campus Montecillo. Texcoco, State, México

**CNIN** Colección Nacional de Insectos, Instituto de Biología, Universidad Nacional Autónoma de México, México City, México

**FSCA** Florida State Collection of Arthropods, Gainesville, Florida, USA

**MNCR** Museo Nacional de Costa Rica, Santo Domingo, Heredia, Costa Rica

**MUCR** Museo de Insectos de la Universidad de Costa Rica, San José, Costa Rica

**MZCR** Museo de Zoología de la Universidad de Costa Rica, San José, Costa Rica

**NMP** National Museum, Prague, Czech Republic

**OUMNH** Oxford University Museum of Natural History, Oxford, United Kingdom

**USNMNH** United States National Museum of Natural History, Washington D. C., USA.

Genitalia of both sexes were dissected by softening the specimens in hot water, removing the abdomen, placing it in a dilute solution of potassium hydroxide until the fat and muscle were dissolved, then rinsing the abdomen and genitalia in clean water and placing the structures in microvials filled with glycerin for later examination.

The anatomy terminology used is based on Gordon (1985) and Gordon et al. (2014); and updated according to Ślipiński (2007). However, the terms for male genitalia position (dorsal, ventral) have been changed to “concave side” or “convex side” because the male genitalia change position and the terms “dorsal side” and “ventral side” can induce error.

The species were assigned to known species groups according to the diagnostic characters described by their authors. The species groups considered as valid are those created by Gordon (1985) and Gordon et al. (2014); a new group was created and described only when the characters of a species or group of species did not match with any known group.

To abbreviate the information, only new species have a complete list of material reviewed, with assignment of holotypes and paratypes. Reviewed material for known species includes only the number of specimens, locations, and the collections where the material is located. In the cases were the type material of a known species was not reviewed, the method of identification of the species is explained.

## Results

Twenty-five species were found, nine of which are new species. The species studied here belong to eight previously described groups and two new groups. Four species could not be identified as members of a group due to the lack of males.

Some species of the groups *lepida* and *dentipes* were previously recorded in North America (Gordon 1985). South American species were not found in Central America, but some species show some affinities with southern fauna. The *dentipes* group was the most abundant in Central America.

### Species key

**Table:** 

1	Elytra orange or yellow without spots	***B. bipartita* Mulsant**
–	Elytra with spots or some marks	**2**
2	Dorsal color mainly black or dark brown	**3**
–	Dorsal color mainly yellow, orange, or pale gray	**17**
3	Elytra with apex of elytral suture dentiform	***B. mimica* sp. nov.**
–	Apex of elytral suture not dentiform	**4**
4	Elytra black with lateral and apical borders yellow	***B. guatemalensis* (Gorham)**
–	Each elytron with one to five spots	**5**
5	Pronotum with two convergent discal spots, each elytron with five pale spots	***B. fenestrata* Gorham**
–	Pronotum without discal convergent spots, elytral maculation variable	**6**
6	Each elytron with five pale spots	***B. octostigma* Mulsant**
–	Elytra with different maculation	**7**
7	Elytra black with a large orange macula covering lateral margin	***B. aurantiapleura* sp. nov.**
–	Elytra with different maculation	**8**
8	Each elytron with three pale spots: one near the scutellar shield and two near the elytral apex	***B. tica* sp. nov.**
–	Elytra with different maculation	**9**
9	Males with setal tuft on center of first ventrite, male genitalia symmetrical	***B. isthmena* sp. nov.**
–	Males without setal tuft on center of first ventrite, male genitalia asymmetrical	**10**
10	Males without humeral spot and without cusps on third ventrite, male genitalia asymmetrical	***B. indubitabilis* Crotch**
–	Males with humeral spot. Third ventrite of male with two cusps, male genitalia asymmetrical.	**11**
11	Fourth ventrite with cusps	***B. barberi* Gordon**
–	Fourth ventrite without cusps	**12**
12	Penis guide of male genitalia with apical hook	**13**
–	Penis guide of male genitalia without apical hook	**15**
13	Apical hook of the penis guide curved and robust	***B. robustihamata* Nestor-Arriola and Toledo-Hernández**
–	Apical hook not curved or robust	**14**
14	Cusps on third ventrite very conspicuous parameres of male genitalia robust and with a tooth on superior margin	***B. dentipes* (Fabricius)**
–	Cusps moderately conspicuous, parameres without tooth	***B. bistripustulata* (Fabricius)**
15	Elytron black without spots, apical margin of elytron red or orange	***B. erythrura* Mulsant**
–	Elytron spotted	**16**
16	Cusps medium sized	***B. subfasciata* Mulsant**
–	Cusps large	***B. truncata* Nestor-Arriola and Toledo-Hernández**
17	Elytra yellow with pale brown lines	***B. aperta* Weise**
–	Elytra with black spots	**18**
18	Each elytron with a large basal macula	***B. papiliona* sp. nov.**
–	Elytra without a large basal macula	**19**
19	Protibia, mesotibia and metatibia toothed, two spots on each elytron	***B. hexaspina* sp. nov.**
–	Mesotibia and metatibia without tooth, maculation variable	**20**
20	Elytra pale with several elongated black spots, tibial tooth small	***B. gorhami* (Weise)**
–	Elytral spots rounded, not elongated	**21**
21	Pronotum mainly black with only lateral borders, anterior border and middle line until center of the disc pale colored. Each elytron with five black spots	***B. nubes* sp. nov.**
–	Pronotum variable, if mainly black with several pale colored projections into the basal black macula. Elytra variable	**22**
22	Elytra yellow with five black spots on each elytron, pronotum with several black small spots	***B. invertita* sp. nov.**
–	Each elytron with two black or brown spots, elytral suture black or with a large dark macula	**23**
23	Male abdomen with central part of ventrites depressed and with dense, long pubescence	***B. cachensis* Gorham**
–	Male abdomen without long pubescence	**24**
24	Dorsal color mainly yellow, orange or pink, sometimes dark with 12 pale spots; male abdomen without modifications	***B. lepida* Mulsant**
–	Dorsal color yellow. Male abdomen with acute tooth in middle of first and second ventrites	***B. dentata* sp. nov.**

### Species accounts

#### *dentipes* group

Abdomen of male with the third ventrite bicuspid, penis guide of male genitalia asymmetrical (Gordon 1985; Gordon et al. 2014).

##### Brachiacantha barberi

Taxon classificationAnimaliaColeopteraCoccinellidae

Gordon

457CE6F6-A937-59ED-8C72-B35CAC3C5385

Brachiacantha barberi Gordon, 1985: 572. Nestor-Arriola and Toledo-Hernández 2019: 548.

###### Material examined.

Material listed in Nestor-Arriola and Toledo-Hernández (2019).

###### Diagnosis.

Length 3.2–3.9 mm. Width 2.6–2.9 mm. Dorsally black with two to three red to orange spots; pronotum mostly black. Third and fourth ventrites bicuspid. Male genitalia asymmetrical, penis guide without apical hook.

###### Distribution.

From the southeast of the USA to Costa Rica.

###### Discussion.

This species is similar to *B. bistripustulata*, but it can be distinguished by its smaller size and the fourth bicuspid ventrite of *B. barberi*.

##### Brachiacantha bistripustulata

Taxon classificationAnimaliaColeopteraCoccinellidae

(Fabricius)

81015BD6-C571-5B37-AFA3-0DBAEF4ED794

Coccinella laevis Thunberg, 1781: 20.
Coccinella bistripustulata Fabricius, 1801: 383.
Brachyacantha bis-tripustulata : Mulsant 1850: 528.
Brachyacantha bistripustulata : Gorham 1894: 188. Casey 1899: 119. Leng 1911: 296. Korschefsky 1931: 203. Blackwelder 1945: 449.
Brachyacantha erythrocephala Gorham, 1894: 188 (non Coccinella erythrocephala Fabricius). Crotch 1874: 211.
Brachyacantha decora Casey, 1899: 119. Bowditch 1902: 206.
Brachyacantha bistripustulata var. guttata Weise, 1885: 231. Leng 1911: 297. Korschefsky 1931: 203.
Brachyacantha bistripustulata var. decora : Leng, 1911: 298. Leng 1920: 213.
Brachyacantha bistripustulata var. minor Leng, 1911: 298. Leng 1920: 213.
Brachyacantha bistripustulata var. obscura Leng, 1911: 299. Korschefsky 1931: 204.
Brachyacantha bistripustulata var. quichiana Leng, 1911: 298.
Brachyacantha bistripustulata ab. decora : Korschefsky 1931: 203.
Brachyacantha bistripustulata ab. minor : Korschefsky 1931: 203.
Brachiacantha decora : Gordon 1985: 561.
Brachiacantha laevis : Pope 1987: 58.
Brachiacantha bistripustulata : Milléo and Almeida 2007: 420. Gordon et al. 2014: 10. Nestor-Arriola and Toledo-Hernández 2019: 539.

###### Material examined.

496 specimens from Costa Rica: Alajuela, Cartago, Heredia and Puntarenas; El Salvador: Ahuachapán, La Libertad and San Salvador; Guatemala: El Quiché, El Progreso, Frontera, Jutiapa, Chimaltenango, Baja Verapaz, Santa Rosa and Zacapa; Honduras: El Paraíso, Tegucigalpa, Atlántida, Colón, Cortés, Comayagua, Francisco Morazán, La Paz, Olancho and Yoro; Panamá: Oeste, Coclé, Chiquirí and Darien (CEAM, CNIN, FSCA, USNMNH, OUNMH, MNCR, MUCR, MZCR).

###### Diagnosis.

Length 3.2–5.4 mm. Width 2.4–4 mm. Dorsally black with three to four orange, yellow or red spots on each elytron, spots sometimes fused. Males with pale-yellow head and pronotal anterior border. Third ventrite bicuspid in males, cusps short, wide and with divergent apex. Male genitalia asymmetrical, penis guide with apical hook.

###### Distribution.

From the South of the USA to Brazil and Peru, including Jamaica, Cuba, Puerto Rico and Dominican Republic.

###### Discussion.

This species is very similar to others in the group, but the shape of the male ventral cusps is different in being wider than long, apically divergent and not strongly pronounced, and is usually enough to differentiate it.

##### Brachiacantha dentipes

Taxon classificationAnimaliaColeopteraCoccinellidae

(Fabricius)

FD7DCC0C-79E7-581D-A16D-42C2A7BAD6AA

Coccinella dentipes Fabricius, 1801: 381. Oliver 1808: 1051. Say 1835: 202.
Brachyacantha dentipes : Mulsant 1850: 525. Crotch 1873: 378. Gorham 1894: 196. Casey 1899: 120. Nunenmacher 1909: 162. Leng 1911: 300. Korschefsky 1931: 204. Wingo 1952: 27. Chapin 1974: 44.
Brachyacantha socialis Casey, 1899: 119. Wingo 1952: 27.
Brachyacantha dentipes socialis : Leng 1911: 301.
Brachyacantha dentipes americana Leng, 1911: 302.
Brachyacantha dentipes var. separata Leng, 1911: 301. Wingo 1952: 27.
Brachyacantha dentipes ab. socialis : Korschefsky 1931: 204.
Brachyacantha dentipes ab. separata : Korschefsky 1931: 204.
Brachiacantha dentipes : Gordon 1985: 564. Flores-Mejía and Salas-Araiza 2004: 14. Nestor-Arriola and Toledo-Hernández 2019: 542.

###### Material examined.

340 specimens from Costa Rica: Alajuela, San José, Guanacaste, Heredia, Puntarenas, and San José; Honduras: El Paraíso and Olancho (FSCA, USNMNH, OUMNH, MNCR, MUCR, MZCR).

###### Diagnosis.

Length 3–4.5 mm, width 2.1–3.2 mm. Three to four spots on each elytron; the spots may be fused. Third male ventrite with large, subtriangular divergent cusps. Male genitalia with wide parameres with strong tubercle on the concave margin; penis guide with elongated apical hook.

###### Distribution.

From the south of the USA to Costa Rica, including the Bahamas.

###### Discussion.

This species is very similar to *B. bistripustulata*, but the male third ventrite has larger cusps and the male genitalia are more robust in *B. dentipes*.

##### Brachiacantha erythrura

Taxon classificationAnimaliaColeopteraCoccinellidae

Mulsant

DD7A5258-E0C1-5EBB-8E87-A67545FD179A

Brachyacantha erythrura Mulsant, 1850: 530. Crotch 1874: 211. Gorham 1894: 187. Leng 1911: 302. Nestor-Arriola and Toledo-Hernández 2019: 545.

###### Material examined.

Material listed in Nestor-Arriola and Toledo-Hernández (2019).

###### Diagnosis.

Length 3.8–3.9 mm, width 2.8–2.9 mm. Dorsally black, head and posterior margin of the elytron red, orange or yellow. Ventral surface light brown to orange. Third ventrite of the male with small cusps. Penis guide of the male genitalia asymmetrical, without apical hook.

###### Distribution.

México and Central America.

###### Discussion.

This species can be confused with some specimens of *B. quadrillum* LeConte, but the third ventrite cusps of *B. erythrura* are much smaller and the male genitalia lack the apical hook.

##### Brachiacantha robustihamata

Taxon classificationAnimaliaColeopteraCoccinellidae

Nestor-Arriola & Toledo-Hernández

91D2D13F-0707-5033-9B52-2C3B82E82F5B

Brachiacantha robustihamata Nestor-Arriola & Toledo-Hernández, 2017: 48.

###### Material examined.

Material listed in Nestor-Arriola and Toledo-Hernández (2017).

###### Diagnosis.

Length 4.5 mm, width 3.2 mm. Oval body. Three spots on each elytron, including an incomplete transversal band. Third ventrite with a small narrow cusp at each side of middle. Apical hook of the penis guide of the male genitalia large and rounded.

###### Distribution.

Guatemala and Costa Rica.

###### Discussion.

This species is very similar to *B. bistripustulata* and other species of the *dentipes* group. The principal difference is the male genitalia with a robust curved hook and male ventral cusps longer than wide. The cusps in the third ventrite are similar to those of *B. blaisdelli* Nunenmacher, but the male genitalia of *B. robustihamata* are distinguished by the curved hook.

##### Brachiacantha subfasciata

Taxon classificationAnimaliaColeopteraCoccinellidae

Mulsant

279C8FCF-49B8-580E-974B-9B625FCA5C96

Brachyacantha subfasciata Mulsant, 1850: 527. Crotch 1874: 211. Gorham 1894: 187. Leng 1911: 302. Leng 1920: 212. Korschefsky 1931: 207.
Brachiacantha subfasciata Gordon, 1985: 574. Nestor-Arriola and Toledo-Hernández 2019: 550.

###### Material examined.

Material listed in Nestor-Arriola and Toledo-Hernández (2019).

###### Diagnosis.

Length 3.8–5 mm, width 2.8–3.3 mm. Oval body. Dorsally black with one to four spots on each elytron. Third ventrite of male with separated cusps connected by a curved emargination. Male genitalia asymmetrical; penis guide almost truncate, without apical hook.

###### Distribution.

From the center of the USA to Honduras.

###### Discussion.

This species is very similar to others of the *dentipes* group, but the male genitalia are different as they lack the apical hook on the penis guide.

##### Brachiacantha truncata

Taxon classificationAnimaliaColeopteraCoccinellidae

Nestor-Arriola & Toledo-Hernández

5E17AAFC-4D25-56F5-82F7-20EA88F973CD

Brachiacantha truncata Nestor-Arriola & Toledo-Hernández, 2017: 45.

###### Material examined.

Material listed in Nestor-Arriola and Toledo-Hernández (2017).

###### Diagnosis.

Length 3.2–5.3 mm, width 2.5–4.2 mm. Male with four spots on the elytron, females with three spots on the elytron. Males with a triangular, dull apex, cusp at each side of the middle line. Male genitalia with penis guide without apical hook, shorter than the parameres, oblique at apex, almost truncate.

###### Distribution.

México and Honduras.

###### Discussion.

The male genitalia with oblique apex and the ventral cusps with blunt apex are enough to distinguish the species from others in the group.

#### *lepida* group

Male genitalia with penis guide asymmetrical, apex obliquely truncate, penis without alae (Gordon 1985). *Brachiacantha lepida* and *B. indubitabilis* were classified in two different monospecific groups by Gordon (1985), distinguished by differences in the male abdomen; however, after Gordon et al. (2014) the male genitalia are almost the only criterion used for group recognition purposes. There are no significant differences between the genitalia of the species, and they are, therefore, classified in the same group.

##### Brachiacantha lepida

Taxon classificationAnimaliaColeopteraCoccinellidae

Mulsant

06BBFBB5-845C-5006-BCFF-08982BB2CDF8

Figures 1 , 2

Brachyacantha lepida Mulsant, 1850: 523. Crotch 1873: 378. Crotch 1874: 210. Gorham 1894: 185. Leng 1911: 324. Leng 1920: 212. Korschefsky 1931: 205.
Brachyacantha duodecimguttata Leng, 1911: 289. **Syn. nov.**

Brachyacantha 12-guttata: Leng 1911: 294. **Syn. nov.**
Brachiacantha lepida : Gordon 1985: 599.

###### Material examined.

271 specimens from Belize: Orange Walk; Costa Rica: Alajuela, Cartago, Heredia, Guanacaste, Limón, San José and Puntarenas; Guatemala: Chimaltenango, El Quiche, Guatemala City, Huehuetenango, Suchitepequez and Totonicapan; Honduras: Atlantida; Panamá: Chiriqui (CEAM, CNIN, FSCA, USNMNH, OUMNH, MNCR, MUCR, MZCR).

###### Diagnosis.

**Figures 1–4.**
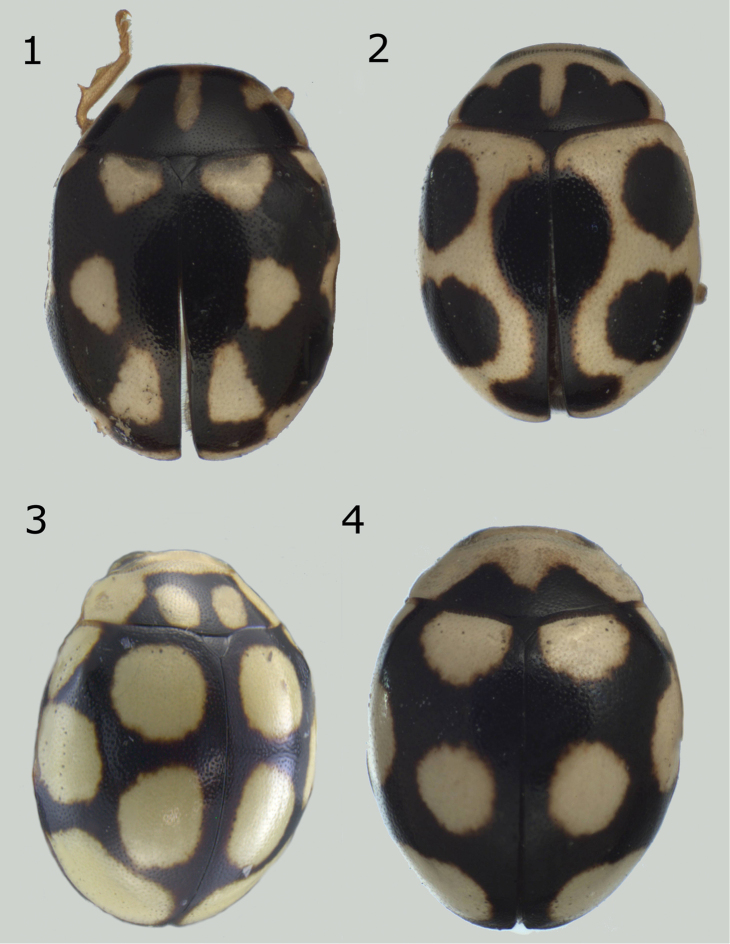
*Brachiacantha* sp. **1***Brachiacantha lepida*, dark habitus (image by Jorge Valdez Carrazco) **2***B. lepida*, common habitus (image by Jorge Valdez Carrazco) **3***Brachiacantha fenestrata*, male **4***Brachiacantha octostigma*, male (image by Jorge Valdez Carrazco).

Length 2.3–3.7 mm, width 1.8–2.8 mm. Oval body. Dorsally pale yellow to pinkish orange except pronotum with a multilobed basal macula; each elytron with 4 black spots, one spot in a sub-humeral position, another in the second half of the elytron, a discal spot on the elytral suture, and a subapical spot on the elytral suture (Figs 1, 2). Ventral color dark brown to light brown. Males with the ventrites V and VI slightly emarginated, not depressed. Male genitalia asymmetrical. Protibia flanged.

###### Distribution.

From southern USA to Panamá.

###### Discussion.

The small size and coloration are usually enough to distinguish this species from other species of the genus. Some specimens from Guatemala display the darker dorsal coloration that was previously described by Leng (1911) as the species *B. duodecimguttata*. The holotype of *B. duodecimguttata* is a female; the genitalia of male specimens with morphology similar to that described by Leng (1911) were dissected and no differences were found from that of *B. lepida*. Therefore, *B. duodecimguttata* should be considered a junior synonym of *B. lepida*.

##### Brachiacantha indubitabilis

Taxon classificationAnimaliaColeopteraCoccinellidae

Crotch

A4DE1A3A-CC7A-5255-B911-827FE74FC13B

Brachyacantha indubitabilis Crotch, 1873: 379. Casey 1899: 120. Leng 1911: 315. Korschefsky 1931: 205.
Hyperaspis triplicans Casey, 1924: 163. Korschefsky 1931: 198. Dobzhansky 1941: 11.
Hyperaspis triplicans microsticta Casey, 1924: 163. Korschefsky 1931: 192. Dobzhansky 1941: 11.
Brachiacantha indubitabilis Gordon, 1985: 596. Flores-Mejía and Salas-Araiza 2004: 14.

###### Material examined.

Costa Rica • 2♀, 6♂; Guanacaste Prov., 20 km N Liberia; 15 Jul 1989; David G. Furth leg. (USNMNH). Guatemala • 1♂; Chicacao; 07 Jul 1949; T. H. Farr leg. (USNMNH).

###### Diagnosis.

Length 3.2–3.7 mm, width 2.5–2.8 mm. Dorsally black, pronotum with lateral and anterior margins yellow, orange, or red; each elytron with three orange or red spots, males and females without humeral spots. Males without ventral cusps. Penis guide of male genitalia asymmetrical.

###### Distribution.

United States of America and México. New record to Costa Rica and Guatemala.

###### Discussion.

This species shows a color pattern that is very common in species of the *dentipes* group; however, it can be recognized because it lacks ventral cusps and lacks humeral spots in males.

#### *buckleyi* group

Penis guide of male genitalia long, slender, apically truncated. Parameres longer than penis guide, very wide at base, narrowed to rounded apex (Gordon et al. 2014).

##### Brachiacantha nubes

Taxon classificationAnimaliaColeopteraCoccinellidae

Nestor-Arriola, Toledo-Hernández & Solís sp. nov.

ED4E49CE-6A78-5D2E-8B15-6E833040B571

http://zoobank.org/53F5C5F1-0FFC-4459-A961-6925D79CB500

Figures 21–24

###### Holotype.

Male, pinned, with genitalia in a separate microvial. Original labels: “Est. Santa Elena, Viejo, Santa Elena, Las Nubes, Prov. San José, COSTA RICA, 1210 m, 23–28 Oct. 1995, col. E. Alfaro, L_S_371750_507800, #6385” (MNCR).

###### Diagnosis.

Dorsal color pale orange with five black spots on each elytron, pronotum orange with a basal black macula covering more than the half of the pronotum.

###### Description.

Male holotype. Length 2.4 mm, width 2 mm; body rounded, convex. Dorsal color pale orange except pronotum with large black macula covering more than the half except the lateral and anterior margins and the middle line in the apical half until the center of the pronotal disk; scutellar shield black; each elytron with five black oval spots: sub-humeral spot large, scutellar spot over the elytral suture near the middle of the elytra, mid-lateral spot on the apical half of the elytra, discal spot on the apical half of the elytra, sub-apical spot over the elytral suture (Fig. 21). Ventral surface with head, prosternum, metaventrite and abdomen dark brown; legs, mouthparts and antennae yellow. Head punctures small, separated by 1½ their diameter, each puncture as large as an eye facet; pronotal punctures larger than head punctures, separated by 1½× their diameter; elytral punctures as large as head punctures, separated by 1½× their diameter; metaventral punctures larger than pronotal punctures, separated by their diameter. Clypeus emarginated. Prosternal carinae not examined. Protibia not flanged, basal tooth small. Basal abdominal ventrite without setal tuft. Abdomen with postcoxal line on basal abdominal ventrite slightly flattened along posterior ventrite margin; ventrite with sparse, long pubescence and small, dense punctures. Ventrites IV–VI depressed and emarginated (Fig. 22). Ventrites II–VI pubescent throughout, punctures fine, dense; third ventrite with a tubercle on each side of central part. Genitalia with penis guide slightly shorter than parameres, symmetrical, sides slightly convergent, slightly wider before apex, apex truncate (Fig. 23); parameres curved, wide at base, narrowed to apex, apex acute, setae arising from the apex (Fig. 24). Penis not examined.

Female. Unknown.

###### Variation.

Unknown.

###### Etymology.

The name refers to the type location.

###### Type locality.

COSTA RICA, San José: Santa Elena de General Viejo, Las Nubes Biological Reserve Ecocampus (Faculty of Environmental Studies, York University), 9°23'08.05"N, 83°36'11.82"W.

###### Distribution.

Costa Rica.

###### Discussion.

This species is easily identifiable by its dorsal coloration. The only species it could be confused with is *B. lepida*; however, the number and arrangement of elytral spots are different in these species as *nubes* has more spots than *lepida*.

##### Brachiacantha fenestrata

Taxon classificationAnimaliaColeopteraCoccinellidae

Gorham

95E70343-C617-5F44-BFA6-04E6A225E35A

Figures 3 , 25–28

Brachyacantha fenestrata Gorham, 1894: 190. Leng 1911: 322.

###### Material examined.

160 specimens from Costa Rica: Alajuela, Guanacaste, Heredia, and Puntarenas; Panamá: Chiquirí (MNCR, MUCR, MZCR).

###### Diagnosis.

Dorsally black or dark brown; pronotum with the anterior angles, the anterior margin and two convergent oval spots on the center of the disc, pale yellow; each elytron has five pale spots (Fig. 3). Male abdomen with ventrites I-V depressed, emarginated, and abundantly pubescent. Male genitalia with the penis guide apically truncate and slightly longer than parameres, symmetrical, with convergent sides (Fig. 25); parameres slender, apex rounded, setae arising from the convex side margin (Fig. 26); penis curved in basal 1/2, apex with small alae, basal capsule without crest, inner arm of basal capsule long and slender (Figs 27, 28).

###### Variation.

In some specimens the elytral spots are fused.

###### Distribution.

Costa Rica and Panamá. The records of México must be confirmed.

###### Discussion.

This species is easily recognizable by the pronotal pale spots.

#### *juanita* group

Male genitalia with penis guide short, evenly, ovately narrowed from base to apex, apex narrowly rounded, sometimes acute; penis with inner arm of basal capsule apically bifid. Female genitalia with spermathecal capsule long, slender, basal ¼ widened, cornu bulbous or narrowed to acute apex (Gordon et al. 2014).

##### Brachiacantha octostigma

Taxon classificationAnimaliaColeopteraCoccinellidae

Mulsant

E7D8E7A8-50DB-56DA-869D-7644C6C400AF

Figures 4 , 29–32

Brachyacantha octostigma Mulsant, 1850: 539. Crotch 1874: 212. Gorham 1894: 188. Leng 1911: 311.

###### Material examined.

Guatemala • 2♀; Totonicapan, V. Sta. María; 1850 m; 19 Jun 1973; Ginter Ekis leg. (USNMNH) Panamá • 1♀; V. Chiquiri; 2000–3000 ft; Champion leg. (USNMNH).

###### Diagnosis.

Oval body. Elytra black, each elytron with five yellow spots. Male abdomen with several ventrites emarginated and depressed. Male genitalia with penis guide wide, shorter than the parameres, not truncate at apex, symmetrical, sides parallel but convergent at apical ⅓ (Fig. 30); parameres curved, wide at base, narrowed to apex, apex acute, setae arising from the apex and the convex side margin (Fig. 29); penis curved in basal ½, apex without alae, basal capsule crested, inner arm of basal capsule long and slender (Figs 31, 32).

###### Distribution.

México and Central America.

###### Discussion.

The coloration of this species is very similar to that of the *ursina* group, but the male genitalia are different, with a shorter penis guide with the apex not truncate. The material was identified by comparison with previously identified as *B. octostigma* by J. Chapin in 1956 (USNMNH), the diagnosis of the male and the male genitalia is based on Mexican specimens of the same series.

##### Brachiacantha bipartita

Taxon classificationAnimaliaColeopteraCoccinellidae

Mulsant

F886EDA4-33A4-5187-A44E-0FF2DE2E287C

Figures 5 , 33–36

Brachyacantha bipartita Mulsant, 1850: 521. Crotch 1874: 211.
Brachyacantha westwoodii Gorham, 1894: 185 (in part). Leng 1911: 305 (in part).
Brachiacantha bipartita : Booth and Pope 1989: 369. Nestor-Arriola and Toledo-Hernández 2016: 826.

###### Material examined.

Costa Rica • 1♂; San José; 1000–1200 m. a. s. l.; Ago 1980; N. L. H. Krauss leg. (USNMNH). Guatemala • 1♀; Antigua; Oct 1965; L. H. Krauss leg. (USNMNH).

###### Diagnosis.

**Figures 5–8.**
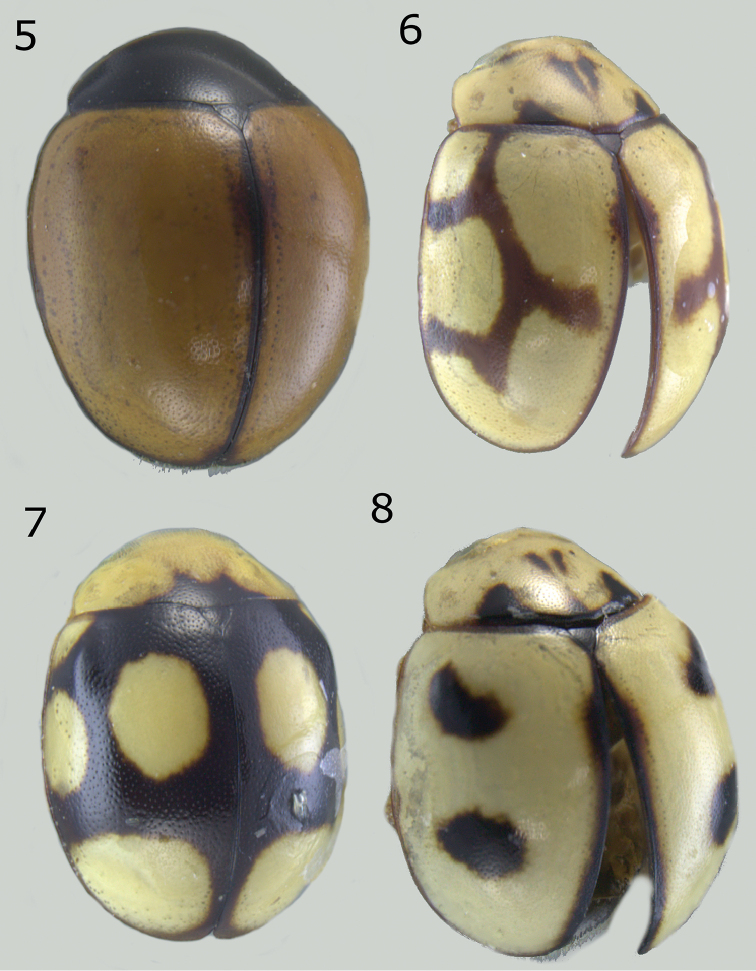
*Brachiacantha* sp. **5***Brachiacantha bipartita*, female **6***Brachiacantha aperta*, female **7***Brachiacantha isthmena* sp. nov. **8***Brachiacantha cachensis*.

Body oval. Elytron orange. Head pale yellow in males, black in females. Males with pronotum black except lateral and anterior margins pale yellow to yellowish white (Fig. 5). Male genitalia with penis guide shorter than parameres, symmetrical, sides convergent at apical ⅓ (Fig. 34); parameres curved, wide at base, narrowed to apex, apex acute, setae arising from the apex and the convex side margin (Fig. 33); penis curved in basal ½, apex with long slender alae (Fig. 36), basal capsule crested, apex long and slender (Fig. 35).

###### Natural history.

This species has been collected on coffee plants (*Coffea* sp.).

###### Distribution.

Mexico and Central America. New record to Costa Rica and Guatemala.

###### Discussion.

This species is easily recognizable by the orange elytra without maculation. The only species similar to *B. bipartita* is *B. westwoodii*, but *B. westwoodii* is much larger and has the pronotum almost entirely orange with only a basal black macula (Nestor-Arriola and Toledo-Hernández 2016). The material was identified following the description of Mulsant (1850) and considering the information of Booth and Pope (1989).

##### Brachiacantha aperta

Taxon classificationAnimaliaColeopteraCoccinellidae

Weise

DF201FF9-5227-5713-9D38-C94DE3DD8D0D

Figures 6 , 37–40

Brachyacantha aperta Weise, 1903: 208. Leng 1911: 323.

###### Material examined.

123 specimens from Costa Rica: Limón, Cartago, and Puntarenas (FSCA, USNMNH, MNCR, MUCR, MZCR).

###### Diagnosis.

Color pale yellow or yellowish white. Pronotum with three or four brown spots. Elytron with a brown-colored branched line pattern (Fig. 6). Ventrites I-VI emarginated and depressed at center, depression becoming wider from the basal ventrite to apical ventrite. Ventrites I-III densely punctate, pubescent within the depression more abundant and longer than at the sides. Male genitalia with parameres and penis guide strongly wide; penis guide as long as parameres, wider at middle, symmetrical, apex rounded (Fig. 40); parameres wide, apex rounded, slightly tuberculated in the concave side margin, with abundant setae at apex and the convex margin, weakly curved (Fig. 39); penis with lateral slender alae at apex (Fig. 38), basal capsule not crested, inner arm slender and long, apex spender and long (Fig. 37).

###### Distribution.

Costa Rica.

###### Discussion.

This species is easily recognizable due to its unique coloration.

##### Brachiacantha dentata

Taxon classificationAnimaliaColeopteraCoccinellidae

Nestor-Arriola, Toledo-Hernández & Solís sp. nov.

14DE6905-D4B9-5914-8188-400A38A704AF

http://zoobank.org/59BC56D7-F3EC-4E43-B641-AFE87908FEF5

Figures 41–45

###### Holotype.

Male, pinned, with genitalia in a separate microvial. Original labels: “San Lorenzo, R. F. Cord., Guanacaste, COSTA RICA, 1050 m, abril 1991, col. C. Alvarado, L-N 287800_427600” (MNCR).

###### Paratypes.

Costa Rica • 1♂; Alajuela, P. N. V. Tenorio, V. Rio Roble, Palmital, “arriba la caliza”; 1000–1100 m; 21 June-16 Sep. 2006; J. A. Azofeifa leg.; malaise trap; L_N_296500_426300, #87178 (MNCR). 1♂; Alajuela, Palmarena, Rio San Lorencito, 5 km N of R. F. Sn Ramón; 900 m; Mar. 1990; Curso Carabidae exped.; 244500-470700 (MNCR). 1♂; Cartago, A. C. Amistad, P. N. Tapantí, Rio Dos Amigos; 1480 m; Mar. 1994; G. Mora, A. Solís and E. Ulate leg; L N 187600_560250, #2782 (MNCR). 1♂; Cartago, Capellades; 2–5 Apr. 1942; Biering leg., on *Carica papaya* L.; det. J. B. Chapin 1991 (MUCR). 1♂; Cartago, P. N. Tapantí; 1450 m; 13–15 May. 2005; Barries, Cate and Nagy leg.; L_N_190920_560811, #86647 (MNCR). 2♂; Cartago, P. N. Tapantí, Quebrada Segunda; 1250 m; Nov. 1992; G. Mora leg.; L-N 194000_560000 (MNCR). 1♂, same data; Dec. 1992 (MNCR). 3♂; same data; ago.1992 (MNCR). 1♂; same data, sep.1992; (MNCR). 2♂; same data; July 1992 (MNCR). 1♂; same data, Oct. 1992 (MNCR). 1♂; same data; July 1992; det. Elena Ulate INBio June 2003 as *B. cachensis* (MNCR). 1♂; Cartago, P. N. Tapantí, Quebrada Segunda; 1300 m; Oct. 1993; G. Mora leg.; L-N 194000_559800, #2286 (MNCR). 1♂; Cartago, Ref. Nac. Fauna Silv. Tapanti; 1250 m; Aug. 1991; F. A. Quesada leg.; L_N_194000_559800 (MNCR). 1♂; Cartago, Turrialba, M. N. Guayabo; 1100 m; 21 June 1994; F. J. Corrales leg.; L N 570000_217400, #3028” (MNCR). 1♂. Cartago, Turrialba, Tayutic, Grano de Oro; 1120 m; Oct. 1993; P. Campos leg.; L_N_200250_595900, #2438 (MNCR). 2♂; Guanacaste, R. F. Cord. Guanacaste, R. San Lorenzo, Tierras Morenas; 1050 m; Dec. 1991; C. Alvarado leg. L-N-287800_427600 (MNCR). 1♂; Guanacaste, Rio San Lorenzo, Tierras Morenas; 1050 m; 8 Mar.- 26 Apr. 1995; G. Rodríguez leg.; malaise trap; L.N. 287800_427600, #4488 (MNCR). 1♂; same data; dic.1992; #1764 (MNCR). 1♂; Puntarenas, A. C. Arenal, Buen Amigo, San Luis Monteverde; 1000–1350 m; May 1994; Z. Fuentes leg.; L N 250850_449250, #2926 (MNCR). 1♂; Puntarenas, R. B. Monteverde; 1520 m; Nov. 1993; N. Obando leg.; L N 253250_449700, #2478 (MNCR). 1♂; Puntarenas, R. B. Monteverde, est. La Casona; 1520 m; Mar. 1994; N. Obando leg.; det. Elena Ulate INBio June 2003 as *B. cachensis*; LN 253250_449700, #2819 (MNCR). 2♂; Puntarenas, R. B. Monteverde, est. Leonel Hernandez; 1600 m; June 1991; E. Bello leg.; L-N-249750_450075 (MNCR). 1♂. Puntarenas, R.B. Monteverde, San Luis; 1040 m; 24 Aug.-15 Sep. 1992; Z. Fuentes leg.; L-N-250850_449250 (MNCR). 1♂; same data; Sep. 1992 (MNCR). 1♂; San José, P. N. Braulio Carrillo; 1600 m; 26 Aug. 1998, C. W. and L. B. O´Brien leg. (FSCA).

###### Diagnosis.

Dorsal color pale yellow with brown or black spots, ventrally brown. Males with a sharpened tubercle or tooth on second ventrite and a sharpened tubercle on each side of the center of the third ventrite. Male genitalia with penis guide wide, convergent at apex; parameres wide at base, rounded at apex; penis with a subtriangular projection near the alae.

###### Description.

Male holotype. Length 3.1 mm, width 2.5 mm; body oval, convex. Dorsal color pale yellow except pronotum with a basal, short and wide brown macula, and a small spot on each side of the center of the disc; scutellar shield brown; elytron with three pale brown marks: sub-humeral and sub-apical spots closely oval, sutural vitta over the elytral suture from near the scutellar shield to the elytron apex (Fig. 41). Ventral surface with prosternum, metaventrite and abdomen brown; legs, mouthparts, and antennae yellow. Head punctures small, separated by their diameter, each puncture slightly larger than an eye facet; pronotal punctures as large as head punctures, separated by their diameter or less; elytral punctures larger than pronotal punctures, separated by their diameter or more; metaventral punctures as large as elytral punctures, separated by more than three diameters. Clypeus straight. Carinae on prosternal process straight, convergent but almost parallel, not joined, larger than half the prosternum. Protibia only slightly flanged with the border almost parallel to tibia, basal tooth small. Basal abdominal ventrite without setal tuft. Abdomen with postcoxal line on basal abdominal ventrite slightly flattened along posterior ventrite margin, ventrite with sparse, long pubescence and small, dense punctures. Ventrite II with a spur in the center at the posterior margin; ventrite III with a spur on each side of the middle line at the posterior margin. Ventrites IV-VI depressed and medially emarginated (Fig. 42). Ventrites II-VI pubescent throughout, punctures fine, dense. Genitalia with penis guide as long as parameres, symmetrical, parallel sided, convergent at apex (Fig. 44); parameres wide basally, narrowed to apex, apex rounded and wide, setae rising from the convex side border and apex (Fig. 43); penis curved in basal ½, apex with small alae and a pair of flattened subtriangular appendages before the alae, basal capsule without crest, inner arm of basal capsule long and slender (Fig. 45).

Female. Unknown. There were several females with the same data as males identified as *B. dentata*, but it was not possible to differentiate them from females of *B. cachensis*, a very similar species of the same region. Males of *B. cachensis* and *B. dentata* have different sexual characters.

###### Variation.

Length 2.6–3.2 mm, width 2–2.5 mm. The pronotal spots can be fused. On some specimens there are two spurs on the third ventrite, but others have only one.

###### Etymology.

From the Latin *dentatus* (= toothed). The name refers to the ventral spurs or teeth.

###### Type locality.

COSTA RICA, Guanacaste: Reserva Forestal Cordillera de Guanacaste, Rio San Lorenzo, 10°36'39.999"N, 84°59'44.9988"W.

###### Distribution.

Costa Rica.

###### Discussion.

This species is externally similar to *B. cachensis* but can be differentiated from it by the dentate ventrites of the male and the male genitalia with the penis guide pointed at apex. It was not possible to distinguish between the females of these species.

#### *tucumanensis* group

Male genitalia with penis guide about as long as parameres, slightly “pinched” laterally at basal ⅓, widened in apical ½, sides rounded to acute apex (Gordon et al. 2014).

##### Brachiacantha aurantiapleura

Taxon classificationAnimaliaColeopteraCoccinellidae

Nestor-Arriola, Solís & Toledo-Hernández sp. nov.

2E2863B4-AA1F-524A-8221-95DE256B9D4C

http://zoobank.org/2899170B-7D2C-4C4D-8003-341C88AB55F8

Figures 46–51

###### Holotype.

Male, pinned, with genitalia in a separate microvial. Original labels: “COSTA RICA, PUNTARENAS, Monteverde, San Luis, 1000–1400 m, Feb. 1994, col. Z. Fuentes, L N 449250_250850, #2615” (MNCR).

###### Paratype.

Costa Rica • 1♀; same data as the holotype; #2771 (MNCR).

###### Diagnosis.

Dorsal color black with the head and the angles of the pronotum yellow in males; both sexes with a large lateral orange macula on each elytron. Dorsal surface with a slightly golden sheen.

###### Description.

***Holotype*.** Male. Length 2.4 mm, width 1.9 mm; body oval-rounded, convex. Dorsal color black except head pale yellow with black clypeus; pronotum with lateral angles and anterior margin pale yellow; elytron with a large oval orange lateral macula, the macula touches the lateral margin and the humeral angle (Fig. 46), the black color shows a slightly golden sheen. Ventral surface with head, prosternum, metaventrite and abdomen dark brown; legs, mouthparts and antennae yellow. Head punctures small, separated by their diameter, each puncture as large as an eye facet; pronotal punctures larger than head punctures, separated by their diameter to 1½× their diameters; elytral punctures larger than pronotal punctures, separated by their diameter; metaventral punctures as large as elytral punctures, separated by 1½× their diameter. Clypeus straight. Prosternal carinae not examined. Protibia not flanged, basal tooth small, sponda extended beyond protibial margin (Fig. 47). Basal abdominal ventrite without setal tuft. Abdomen with postcoxal line on basal abdominal ventrite curved, touching the posterior ventrite margin, ventrite with sparse, long pubescence and small, dense punctures. Ventrites II-IV with a tubercle on each side of middle, each pair of tubercles more separated than the anterior ventrite; ventrites V and VI flattened and emarginated (Fig. 48). Ventrites II-VI pubescent throughout, punctures fine, dense. Genitalia with penis guide as long as parameres, symmetrical, sides slightly convergent, slightly wider before apex, apex acute (Fig. 50); parameres curved, narrowed to apex, apex acute, setae arising from convex side and the apex (Fig. 49); penis curved in basal ½, apex without alae, apex with a large apical ampulla, basal capsule without crest, inner arm of basal capsule small and subtriangular (Fig. 51).

Female. Length 2.5 mm, width 1.9 mm. Similar to male except head, pronotum and basal part of femurs black; abdomen not modified. Genitalia not examined.

###### Variation.

The only known variation is shown by the female paratype.

###### Etymology.

From the Latin word *aurantius* (= orange) and *pleurón* (= sides). The name refers to the dorsal color pattern.

###### Type locality.

COSTA RICA, Puntarenas: Monteverde, San Luis, 10°17'11"N, 84°48'5"W.

###### Distribution.

Costa Rica.

###### Discussion.

This species is easily recognizable by its dorsal coloration and the gold sheen on the dorsal surface. The species is very similar to *Hyperaspis panzosae* Gorham, but the specimen described by Gorham is a female and the author described the specimen having black legs (however, the figure in Gorham’s table has orange legs instead), while the female described here has only the basal half of femurs black; the author did not describe any sheen for *H. panzosae*, while *aurantiapleura* has a gold sheen on the dorsal surface. There are several species of *Hyperaspis* with similar coloration to *panzosae* like *H. excelsa* Fall and *H. cruenta* LeConte, so that without examining the original material there is no evidence to assume *panzosae* as *Brachiacantha* instead of *Hyperaspis*; therefore, we decided to maintain *aurantiapleura* as a separate species.

##### Brachiacantha isthmena

Taxon classificationAnimaliaColeopteraCoccinellidae

Nestor-Arriola, Toledo-Hernández & Solís sp. nov.

B36B9168-5F1C-54C8-8338-7237858D9C12

http://zoobank.org/0BFCD30B-0966-46DA-ABB0-2343719E3781

Figures 7 , 52–55

###### Holotype.

Male, pinned, with genitalia in a separate microvial. Original labels: “PANAMÁ, Cerro Campana, 3000’. July 29 1970, H. and A. Howden” (USNMNH).

###### Paratypes.

Costa Rica • 1♂; Limón, Res. Biol. Hitoy Cerere; 100 m; 6–16 May 1992; G. Carballo leg.; L-N 184200_643300 (MNCR). 1♂; Limón, Valle de la Estrella, R. B. Hitoy Cerere; 100 m; 04 Mar. 1994; G. Carballo leg.; L-N 643400_184600, #2889” (MNCR). Panamá • 1♂; same data as the holotype; 31 July 1970 (USNMNH). 1♂; Pr. Cerro Campana; 8°40'N, 79°56'W, 850 m; 14 July 1971; H. A. Hespenheide leg. (USNMNH). 1♂, same data, 10 Feb. 1973; W. Bivin leg. (USNMNH).

###### Diagnosis.

Dorsal color black or dark brown; pronotum almost completely yellow; each elytron with three or four yellow spots. Male abdomen with a setal tuft on the first ventrite and several ventrites emarginated and depressed.

###### Description.

***Holotype*.** Male. Length 3 mm, width 2.2 mm, body oval, dorsally convex. Dorsal color black except head pale yellow; pronotum pale yellow with only a basal black macula; each elytron with four yellow spots in humeral, discal, mid-lateral and sub-apical positions (Fig. 7). Ventral surface with head, prosternum, metaventrite and abdomen dark brown; legs, mouthparts and antennae yellow. Head punctures small, separated by their diameter, each puncture as large as an eye facet; pronotal punctures larger than head punctures, separated by their diameter; elytral punctures as large as pronotum punctures, separated by their diameter; metaventral punctures as large as elytral punctures, separated by their diameter. Clypeus emarginate. Carinae on prosternal process straight, convergent, joined near the anterior prosternal border, larger than half prosternum. Protibia not flanged, basal tooth small. Basal abdominal ventrite with setal tuft at center. Abdomen with postcoxal line on basal abdominal ventrite slightly flattened along posterior ventrite margin, ventrite with sparse, long pubescence and small, dense punctures. Ventrites II-VI depressed and emarginated, punctures fine, dense. Genitalia with penis guide as long as parameres, symmetrical, slender, sides slightly pinched at basal half, widened in the apical third, apex acute (Fig. 53); parameres slender, basally wide, narrowed to apex, apex rounded, setae rising from the convex side margin and apex (Fig. 52); penis robust, strongly curved in basal half, widened in apical third with a narrowing before apex, alae short and slender, basal capsule without crest, inner arm long, parallel sided, rounded at apex (Figs 54, 55).

Female. Unknown.

###### Variation.

Unknown.

###### Etymology.

The name refers to the Panamá isthmus.

###### Type locality.

PANAMÁ, Panamá Oeste: Parque Nacional Altos de Campana, Cerro de la Campana, 8°42'49.4"N, 79°57'10.24"W.

###### Distribution.

Costa Rica and Panamá.

###### Discussion.

The coloration of the species is similar to that of the *dentipes* group of species, but *B. isthmena* lacks the ventral cusps of the male, instead it shows a setal tuft on the first ventrite.

#### *ursina* group

Abdomen of male with the 5^th^ ventrite modified; penis guide of male genitalia symmetrical, apically truncate (Gordon 1985).

##### Brachiacantha guatemalensis

Taxon classificationAnimaliaColeopteraCoccinellidae

(Gorham) comb. nov.

80AC62FC-D136-5EDB-B356-671BBD010D61

Figures 9 , 56–58

Hyperaspis guatemalensis Gorham, 1894: 200, tab. XI, fig. 10.

###### Material examined.

Guatemala • 1♂; Yepocapa; 01 May 1948; H. T. Palmat leg. (USNMNH).

###### Diagnosis.

**Figures 9–12.**
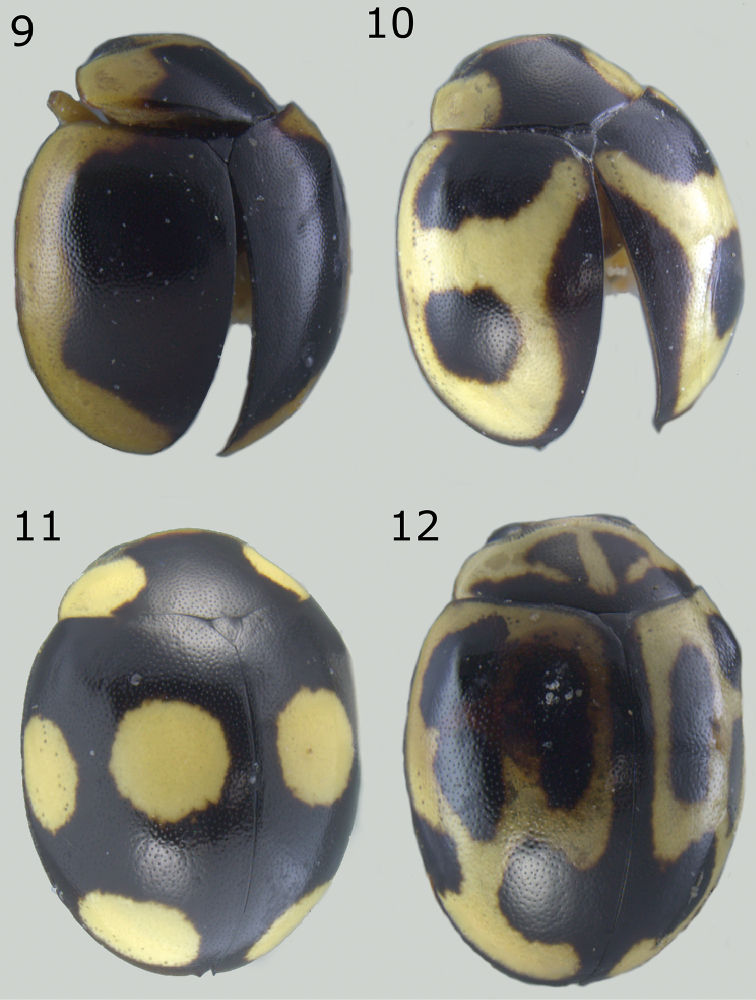
*Brachiacantha* sp. **9***Brachiacantha guatemalensis* comb. nov., male **10***Brachiacantha papiliona* sp. nov., female **11***Brachiacantha mimica* sp. nov., female **12***Brachiacantha gorhami* comb. nov., female.

Length 3 mm, width 2.4 mm; body rounded, convex. Dorsal color black, pronotum with lateral angles and anterior border yellow, each elytron with an irregular yellow macula covering from the humeral angle to the posterior border (Fig. 9). Ventral surface with head, prosternum and metaventrite black; abdomen yellow except the center of ventrites I-III black; legs, mouthparts, and antennae yellow. Carinae on prosternal process parallel, convergent near the anterior margin of prosternum, not joined. Protibia not flanged, basal tooth small. Ventrites I-IV truncate at apex; ventrites V and VI emarginated and depressed. Male abdomen with several ventrites truncate at center. Genitalia with penis guide as long as parameres, symmetrical, sides slightly convergent, apex truncate (Fig. 58); parameres parallel sided, apex rounded, setae rising from the apex border long and curved; penis curved in basal ½, apex with subtriangular alae, basal capsule not crested, inner arm of basal capsule long and slender (Figs 56, 57).

Female. Unknown.

###### Variation.

Unknown.

###### Distribution.

Guatemala.

###### Discussion.

This species is easily identifiable by the dorsal black coloration with elytral and pronotal margins yellow. The specimen examined was identified following the description and illustrations of Gorham (1894) as *Hyperaspis guatemalensis*. Nevertheless, this species has all the diagnostic characters of *Brachiacantha*, including the tibial tooth, lack of large abdominal pores and eye emarginated by the eye canthus; in addition, there are several species of *Hyperaspis* with coloration similar to *H. limbalis* Casey and *H. caseyi* Gordon, but the individual examined presents all the external characteristics, size and distribution of *H. guatemalensis*. Therefore, a new combination is proposed. Several species described by Gorham have been reassigned into other genera due taxonomic revisions (Gordon 1981); about the genus *Hyperaspis* there are several examples: *H. pauperula* was reassigned to *Calloeneis*, *H. adelaia* is considered now as a synonym of *Cyrea tessulata* (Mulsant) and *H. terminata* was reassigned to *Serratitibia* (Canepari et al. 2016; Gordon 1981; Gordon et al. 2013). According the present evidence *H. guatemalensis* should be reassigned to *Brachiacantha*, but an examination of the type material is needed to ensure the taxonomic identity with the material examined here.

#### *invertita* group, new group

Male genitalia with penis guide with sides slightly convergent, almost parallel; apex almost truncate with a triangular projection (Fig. 58). Parameres slender, apically narrowed (Fig. 59).

##### Brachiacantha invertita

Taxon classificationAnimaliaColeopteraCoccinellidae

Nestor-Arriola, Toledo-Hernández & Solís sp. nov.

477D7086-0D1E-55ED-819E-0194624AC020

http://zoobank.org/2665461E-6DF3-4ACF-B4E7-D00FA2BEBFBC

Figures 59–64

###### Holotype.

Male, pinned, with genitalia in a separate microvial. Original labels: “COSTA RICA, PUNTARENAS, A. C. Arenal, Buen Amigo, San Luis, 1000–1350 m, May 1994, col. Z. Fuentes, L N 250850_449250, #2926” (MNCR).

###### Paratypes.

Costa Rica • 1♂; Cartago, Birrisito; 1400 m; 07 Aug. 1980; D. R. Whitehead leg. (USNMNH). 1♀; Cartago, Turrialba, Tayutic, P. N. Barbilla, Sector Cerro Tigre; 1500–1600 m; 22 June-22 July 2001; W. Arana leg.; malaise trap; L_N_211700_602500, #64679 (MNCR); 1♀; Cartago, P. N. Tapantí, Quebrada Segunda; 1250 m; Oct. 1992; G. Mora leg.; L-N 194000_560000 (MNCR). 1♀; Guanacaste, R. F. Cord. Guanacaste, Rio San Lorenzo, Tierras Morenas; 1050 m; Dec. 1991; C. Alvarado leg.; L-N 287800_427600 (MNCR). 2♂; same data; ene.1992 (MNCR). 1♀; Guanacaste, Est. Cacao, SW side Volcán Cacao; 1000–1400 m; Apr. 1988; GNP. Biodiv. Survey exped.; 323300_375700 (MNCR). 1♂; same data; malaise trap; 1988–1989 (MNCR). 1♀, 1♂; Guanacaste, A. C. Guanacaste, Sect. Las Pailas; 800 m; 6–26 June 1994; K. Taylor leg.; L N 309500_389500, #3063 (MNCR). 1♀; Guanacaste, Tierras Morenas; 650 m; 8–10 Feb 1994; Z. Fuentes leg.; L S 283950_424500, #2616 (MNCR). 1♀; Guanacaste, P. N. Rincon de la Vieja, Est. Las Pailas; 800 m; 10–21 Nov. 1993; M. A. Zumbado and D. G. García leg.; L N 306300_388600, #2559 (MNCR). 2♀, 6♂; Guanacaste, sect. Las Pailas, 4.5 km SW Rincón de la Vieja volcano; 800 m; 12 Apr.-4 May 1995; K. Taylor leg.; L_N_306300_388600, #6194 (MNCR). 1♂; Guanacaste, Z. P. Tenorio, Rio San Lorenzo, Tierras Morenas; 1050 m; Aug. 1992; G. Rodriguez leg.; L-N 287800_427600 (MNCR). 1♀; same data, Oct. 1992 (MNCR). 1♂; Guanacaste, Z. P. Tenorio, Rio San Lorenzo, Tierras Morenas; 1050 m; 28 Mar.-21 Apr. 1992; M. Segura leg.; L-N 287800_427600 (MNCR). 1♀; Puntarenas, Monteverde, San Luis; 1000–1350 m, Feb. 1994; Z. Fuentes leg.; L-N 449250_250850, #2615 (MNCR). 1♀, 1♂; Puntarenas, Monteverde, San Luis; 1040 m; 24 Aug.-15 Sep. 1992; F. A. Quesada leg.; L-N 250850_449250 (MNCR). 1♀; Puntarenas, R. B. Monteverde, est. La Casona; 1520 m; July 1992; N. Obando leg.; L N 253200_449700 (MNCR). 1♀; San José, Moravia, San Jeronimo; 1400 m; 05 Dec. 2008; A. S. Saenz leg.; L N 222050_535800, #95353 (MNCR).

###### Diagnosis.

Dorsal color yellow with black spots. Male abdomen with several ventrites depressed and emarginate. Penis guide of male genitalia with an apical triangular projection, penis with long slender alae.

###### Description.

***Holotype*.** Male. Length 3.6 mm, width 2.0 mm; body oval, convex. Dorsal color yellow except pronotum, with a small basal black spot on each side of the disk center, a small black basal spot and a black subquadrate spot on each side at ½ from the lateral margin; scutellar shield black; each elytron with five black spots: scutellar spot slightly oval, sub-humeral spot large and oval, mid-lateral spot at the apical half of the elytron, discal and sub-apical spots rounded, the spots do not touch the elytral margin, the elytral suture or the anterior border (Fig. 59). Ventral surface with head, prosternum, metaventrite and abdomen black; legs, mouthparts, and antennae yellow. Head punctures small, separated by their diameter, each puncture slightly larger than an eye facet; pronotal punctures larger than head punctures, separated by their diameter; elytral punctures as large as pronotal punctures, separated by their diameter; metaventral punctures larger than elytral punctures, separated by more than three diameters at center, about one diameter on sides. Clypeus straight. Carinae on prosternal process curved, divergent, not joined, larger than half the prosternum. Protibia not flanged, basal tooth small. Basal abdominal ventrite without setal tuft. Abdomen with postcoxal line on basal abdominal ventrite slightly flattened along posterior ventrite margin; ventrite with sparse, long pubescence and small, dense punctures. Ventrites III-VI depressed and emarginated (Fig. 60). Ventrites II-VI pubescent throughout, punctures fine, dense; third ventrite with a tubercle on each side of middle. Genitalia with penis guide as long as parameres, symmetrical, sides slightly convergent, apex almost truncated but with a triangular appendage (Fig. 61); parameres narrowed to apex, apex acute, setae rising from the convex side border (Fig. 62); penis curved in basal ½, apex with long slender alae, basal capsule crested, inner arm of basal capsule long and slender (Figs 63, 64).

Female. Similar to male except abdominal characters. Genitalia not examined.

###### Variation.

Males. Length 2.9–3.1 mm, width 2.2–2.7 mm. Females. Length 2.6–3.8 mm, width 2–2.9 mm.

###### Etymology.

From the Latin *inversos* (= invert). The name refers to the dorsal color pattern, yellow with five black spots, the inverse of that of many *Brachiacantha* species (black with five yellow spots).

###### Type locality.

Costa Rica, Puntarenas: Monteverde, San Luis, 10°17'11"N, 84°48'5"W.

###### Distribution.

Costa Rica.

###### Discussion.

This species is easily identifiable by the dorsal coloration, yellow with black spots.

#### *cachensis* group, new group

Male genitalia with penis guide “guitar” shaped, parallel sided at basal ⅓, widened at 2/3, apically emarginated; parameres subtriangular, rounded at apex.

##### Brachiacantha cachensis

Taxon classificationAnimaliaColeopteraCoccinellidae

Gorham

A6CFA797-5944-5522-9BAC-39A208CE4AB4

Figures 8 , 65–68

Brachyacantha cachensis Gorham, 1894: 190. Leng 1911: 322.

###### Material examined.

71 specimens from Costa Rica and Panamá (FSCA, USNMNH, MNCR, MUCR, MZCR). Costa Rica: Alajuela, Cartago, Guanacaste and Puntarenas. Panamá • 1♀; Chiriqui Prv., cont’l Divide trail; 04 Jul 1997; Wappes and Morris leg.; *Brachiacantha* sp. R. Gordon 1998 (USNMNH). 1♀, 1♂; Chiriqui Prv., cont’l Divide trail; 04 Jul 1997; Wappes and Morris leg. (USNMNH). 1♀, 1♂; Chiriqui Prv., cont’l Divide trail; 08 Jul 1997; J. Huether leg. (USNMNH). 1♀; Chiriqui Prv., “Finca La Suiza”; 07 Jul 1997; J. Huether leg. (USNMNH).

###### Diagnosis.

Dorsal color pale yellow to gray with brown or black spots. Ventrites I-VI depressed and emarginated, ventrites I-III with abundant and longer pubescence in the depressed part. Genitalia with penis guide slightly longer than parameres, symmetrical, basal ⅓ with almost straight sides, abruptly wide at apical 2/3 (Fig. 65); parameres wide basally, narrowed to apex, apex rounded (Fig. 66); penis curved in basal ½, widened at the apical ⅓, narrowed before apex, alae rounded, basal capsule with small crest, inner arm of basal capsule long and slender with parallel sides (Figs 67, 68).

###### Variation.

The basal macula over pronotum can be disrupted, forming a macula over the posterior margin with a pair of small spots at the center of the pronotum. In some specimens the sub-humeral spot is half-moon shaped.

###### Distribution.

Costa Rica and Panamá. New record to Panamá.

###### Discussion.

This species is similar to *B. dentata*, but the males can be differentiated by the lack of a ventral tooth and having the central part of the ventrites depressed with abundant pubescence. Within the groups defined by Gordon et al. (2014) for the South American species based on the genitalia of the male, this species does not correspond to any of them, due to the emarginated apex of the penis guide. The material was identified following the descriptions of Gorham (1894) and the keys and illustrations of Leng (1911).

##### Brachiacantha hexaspina

Taxon classificationAnimaliaColeopteraCoccinellidae

González, Větrovec & Nestor-Arriola sp. nov.

21F5D84C-CE4E-51A9-B562-A61B3A3E0D84

http://zoobank.org/27061E37-3AB4-4ECE-BD4A-B986BD822487

Figures 13–20

###### Holotype.

Male, pinned, with genitalia in a separate microvial. Original labels: “PANAMÁ: Veraguas, 12.IX.2017, Santa Fé env., Alto de Piedra-Cerro Tute, 08°30.8'N, 81°07.2'W, 850–1360 m, lower montane forest, individual collecting, Fikàček, Seidel and Sekerka, lgt.” (NMP) (1♂).

###### Paratypes.

Costa Rica • 1♀; Alajuela, P. N. V. Tenorio, Rio Robles, Palmital, “arriba la Caliza”; 1000–1100 m; 21 June-16 Sep.; J. A. Azofeifa leg.; malaise trap; LN296500_426300, #87178 (MNCR). 1♀; Limón, Res. Biol. Hitoy Cerere, est. Hitoy Cerere; 100 m; Oct. 1992; G. Carballo leg.; L-N 184600_643300 (MNCR). 1♀; Limón, Valle de la Estrella, R. B. Hitoy Cerere, send. Espavel; 560 m; 18 Sep.-5 Oct. 2003; B. Gamboa, E. Rojas and W. Arana leg.; malaise trap; L-S 401200_569800, #75488 (MNCR). 1♀; Puntarenas, Golfito, Est. Agujas, Cerro Rincon; 645–745 m; 15 Apr.–15 May 2000; A. Azofeifa leg.; malaise trap; L_S_275500_521950, #56675, sp#106 (MNCR).

###### Diagnosis.

Dorsal color yellow with two black spots in each elytron. Pro-, meso-, and metatibia with external tooth. Male with ventrites II-V emarginate and depressed, forming a longitudinal fossa on abdomen. Male genitalia with penis guide symmetrical, apically notched, penis with a strong curvature at apical 1/6 toward external side.

**Figures 13–20.**
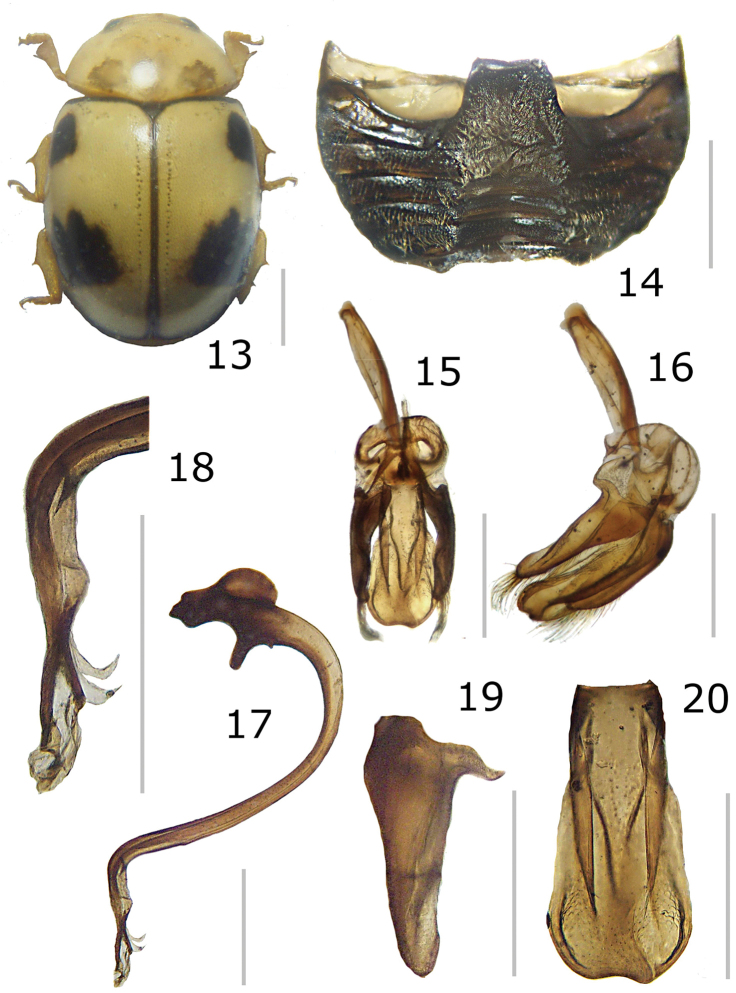
*Brachiacantha hexaspina* sp. nov. male **13** Dorsal view **14** Abdomen **15** Tegmen **16** Tegmen lateral view **17** Penis **18** Penis apex **19** Paramere **20** Penis guide. Scale bar: 1 mm, except **15–20** 0.5 mm

###### Description.

***Holotype*.** Male. Length 4 mm, width 2.9 mm; body oval, slightly elongate, convex. Dorsal color yellow except pronotum with two basal, irregular light brown transparent spots, roughly round, diameter approximately 2/5 the length of pronotum; scutellar shield dark brown; elytron with two black spots: humeral spot irregular and slightly subquadrate, sub-apical spot large, twice the size of the humeral spot, subquadrate and with the basal side trilobed, closer to the lateral edge than to the suture; elytral suture and anterior margin black, lateral margin black until apical ⅓ (Fig. 13). Ventral surface yellow with head and prosternum brown; meso-, metasternum and abdomen dark brown; mouthparts and antennae yellow, mandibular apex black; legs yellow, trochanters light brown. Head punctures small, separated by less than half their diameter, each puncture larger than twice an eye facet; pronotal punctures as large as head punctures, separated by half to one diameter, more compact towards the edges; elytral punctures as large as pronotum punctures, separated by two diameters, irregular lines of large and apparently black dots on the anterior margin of elytra, the basal half of the lateral margin and each side of the elytral suture, the lines are separated from the elytral margins and the elytral suture by approximately the scutellar shield length; metaventral punctures as large as elytral punctures, separated by 1½× their diameters. Clypeus straight. Carinae on prosternal process straight, larger than half prosternum, convergent to prosternal anterior border without reaching it, not joined. Protibia flanged, basal tooth large, curved, as large as protibial width; mesotibia not flanged, basal tooth as large as protibial tooth; metatibia not flanged, basal tooth shorter than protibial tooth. Basal abdominal ventrite without setal tuft. Abdomen with postcoxal line on basal ventrite slightly flattened along posterior ventrite margin, slightly recurved towards the anterior margin at the external ⅓, without reaching the lateral or anterior borders; all ventrites with short and sparse pubescence at sides, more long and less sparse from I–V; ventrite punctures variable, fine and dense, larger towards the lateral and posterior edges. Ventrites II–V emarginated and depressed at middle, ventrite VI flattened, slightly depressed centrally, posterior margin emarginated. Ventrites III-IV with a tubercle at each side of the emargination, II and V only with a slight convexity. Ventrites I and II with long, bushy pubescence at middle, III–V glabrous at the middle (Fig. 14). Genitalia with penis guide longer than parameres, symmetrical, wide, basal ⅓ with almost straight sides, abruptly wide at apical ⅔, wider at apex, sides of the wide part slightly emarginated, apex slightly emarginated (Figs 15, 20), apex sinuous in lateral view (Fig. 16); parameres wide basally, narrowed to apex, apex rounded, setae rising from the apex border (Figs 16, 19); penis curved in basal ½, bended forming a right angle at the apical 1/6, narrowed before apex, alae narrowed apically (Fig. 18), basal capsule crested, heavily sclerotized, inner arm of basal capsule perpendicular to penis, long and slender with parallel sides, external arm in the direction of the tube, twice as long as wide, ending in a short projection (Fig. 17).

Female. Similar to male except the abdomen characters; ventral surface with head, prosternum, metasternum and abdomen orange; legs, mouthparts, and antennae yellowish orange. Mesotibial basal tooth smaller than protibial tooth; metatibial basal tooth shorter and wider than mesotibial tooth. Genitalia not examined.

###### Variation.

Length 3.6 to 4.6 mm. Width 2.8–3 mm.

###### Etymology.

The name derives from Greek *hexa* (= six) and Latin *spine* (= thorn), referring to the six teeth of the tibiae, long and curved like rose thorns.

###### Type locality.

PANAMÁ, Veraguas: Santa Fé, Alto de Piedra, 08°30.8'N, 81°07.2'W.

###### Distribution.

Costa Rica and Panamá.

###### Discussion.

This species is easily identifiable by the pale dorsal coloration and the toothed pro-, meso- and metatibia. The presence of tibial spines on the middle and hind legs is a feature not previously observed in the genus. According to the groups defined by Leng (1911), this species belongs to group V, characterized by the presence of an abdominal canal that covers ventrites II-V, within which only *B. cachensis* Gorham has a similar design but whose male genitalia are different. Within the groups defined by Gordon et al (2014) for the South American species based on the genitalia of the male, this species does not correspond to any of them, due to the notch at the apex of the penis guide. Despite these peculiarities, the similarities in the abdominal ventrite modifications with other species from Central America, the lack of abdominal primary pores, and the presence of protibial tooth make it prudent to keep it in *Brachiacantha*.

Species not associated with males.

##### Brachiacantha papiliona

Taxon classificationAnimaliaColeopteraCoccinellidae

Nestor-Arriola, Toledo-Hernández & Solís sp. nov.

D1C0B676-0BCC-53BC-BE32-7698BD82C88A

http://zoobank.org/54CECBE8-2235-445B-A88B-F2DE8C096315

Figure 10

###### Holotype.

Female, pinned, with genitalia in a separate microvial. Original labels: “PANAMÁ, Chiquiri. Prov., cont’l Divide trail, 04-VII-1997, col. Wappes and Morris” (USNMNH) (1♀).

###### Paratypes.

Costa Rica • 1♀; Guanacaste, Est. Cacao, SW side Volcan Cacao, 1000–1400 m; 1988–1989; GNP. Biodiv. Survey exped.; malaise trap; 323300_375700 (MNCR). 1♀; Puntarenas, Monteverde, San Luis; 1000–1350 m; Feb. 1994; Z. Fuentes leg.; L N 449250_250850, #2615 (MNCR).

###### Diagnosis.

Dorsal color pale yellow with black macula. Elytron with part of the elytral suture black, a sub-apical black spot and a large black spot on the anterior margin.

###### Description.

***Holotype*.** Female. Length 3 mm, width 3.6 mm; body oval-rounded; convex. Dorsal color pale yellow except pronotum with the central part black; scutellar shield black; each elytron with three black spots: a macula over the elytral suture, a large oval spot at the center of the second half of the elytron and an irregular macula over the anterior margin of the elytron (Fig. 10). Ventral surface with head, prosternum, meso- and metasternum and abdomen black; legs, mouth parts and antennae yellow. Head punctures small, separated by their diameter or less, each puncture as large as an eye facet; pronotal punctures larger than head punctures, separated by less than their diameter; elytral punctures as large as pronotal punctures, separated by their diameter; metaventral punctures larger than elytral punctures, separated by 1½× their diameter to two diameters. Elytral suture with apex dentiform. Clypeus straight. Carinae on prosternal process convergent, joined, longer than half prosternum. Protibia not flanged, basal tooth small. Basal abdominal ventrite without setal tuft. Abdomen with postcoxal line on basal abdominal ventrite slightly flattened along posterior ventrite margin, ventrite with sparse, long pubescence and small, dense punctures. Ventrites II-VI pubescent throughout, punctures fine, dense. Genitalia with two curved sclerotized arms at bursa copulatrix; apical strut long, flattened laterally, straight sided, slightly widened at apex; spermathecal capsule slender, widened and rounded at cornu.

Male. Unknown.

###### Variation.

Unknown.

###### Etymology.

The name refers to the basal elytral spot in the shape of butterfly wings.

###### Type locality.

Panamá, Chiriqui: Hornito, 8°44'50"N, 82°13'84"W.

###### Distribution.

Panamá and Costa Rica.

###### Discussion.

This species resembles *B. hazel*Gordon et al. (2014) for the basal macula on the elytron, but the color pattern is different in lacking the sub-apical spot over the sutural margin. Gordon and collaborators did not describe the female of *B. hazel*, but the missing sub-apical elytral spot is not associated with sexual dimorphism in *Brachiacantha* species. Another remarkable character of *B. papiliona* is the presence of a tooth at the apex of the elytral suture, a rare character among Coccinellidae.

##### Brachiacantha tica

Taxon classificationAnimaliaColeopteraCoccinellidae

Nestor-Arriola, Toledo-Hernández & Solís sp. nov.

F7B73900-624C-5E39-A84B-9E769E45EEC4

http://zoobank.org/8180E24F-BD61-4463-80C1-0E6D19F8DFC4

Figures 69–70

###### Holotype.

Female, pinned. Original label: “COSTA RICA, PUNTARENAS, Península de Osa, Rancho Quemado, 200 m, Sep. 1992, col. A. Marín, L-S 292500_511000” (MNCR).

###### Paratypes.

Costa Rica • 1♀; Puntarenas, same data than the holotype; 10 Sep.-10 Oct. 1993 (MNCR). 1♀; same data as holotype; Dec. 1992; F. A. Quesada leg.; (MNCR). 2♀; Puntarenas, P. N. Corcovado, send. Rio Claro; 1991; L-S 508300_270500 (MNCR). 1♀; Puntarenas, P. N. Corcovado, Est. Sirena; 0–100 m; Mar.-June 1991; L-S 270500_508300 (MNCR). 1♀; same data, 17 June-4 Sep. 1991; malaise trap; (MNCR).

###### Diagnosis.

Dorsal color black; pronotum anterior angles pale yellow; each elytron with three pale yellow spots, one near the scutellar shield and two near the apex, no humeral or mid-lateral spots. Sponda large and conspicuous.

###### Description.

***Holotype*.** Female. Length 2.3 mm, width 1.7 mm; body oval, convex. Dorsal color black except head pale yellow; pronotum with lateral angles pale yellow; elytron with three pale yellow oval spots: scutellar shield spot small, small discal spot in the apical half of elytra, sub-apical spot large and oval (Fig. 69). Ventral surface with head, prosternum, metaventrite and abdomen dark brown; legs, mouthparts and antennae yellow. Head punctures small, separated by their diameter, each puncture as large as an eye facet; pronotal punctures larger than head punctures, separated by their diameter; elytral punctures as large as those on pronotum, separated by their diameter; metaventral punctures larger than elytral punctures, separated by their diameter to two diameters. Clypeus slightly emarginated. Carinae on prosternal process straight, larger than half prosternum, convergent, joined near the anterior margin of prosternum but not touching it. Protibia not flanged, basal tooth small, sponda obvious (Fig. 70). Basal abdominal ventrite without setal tuft. Abdomen with postcoxal line on basal abdominal ventrite curved, only touching the posterior ventrite margin, ventrite with sparse, long pubescence and small, dense punctures. Ventrites II-VI pubescent throughout, punctures fine, dense. Genitalia not examined.

Male. Unknown.

###### Variation.

Length 2.1–2.6mm. Width 1.5–1.8 mm.

###### Etymology.

The species name is a popular name for the inhabitants of Costa Rica.

###### Type locality.

COSTA RICA, Puntarenas: Península de Osa, Rancho Quemado, 8°40'54.88"N, 83°33'33.81"W.

###### Distribution.

Costa Rica.

###### Discussion.

Although having three spots on each elytron is common in several *Brachiacantha* species, the particular pattern of *B. tica*, with one spot in the basal half of the elytron and two in the apical half, is not shared with any other species of the genus.

##### Brachiacantha mimica

Taxon classificationAnimaliaColeopteraCoccinellidae

Nestor-Arriola & Toledo-Hernández sp. nov.

3B7F7C5B-99A4-5512-8D11-863865CCE06D

http://zoobank.org/E8115FF9-80D8-45AF-BF28-19C2B784D002

Figure 11

###### Holotype.

Female, pinned. Original labels: “GUATEMALA, ANTIGUA, 1500–1600 m, 01-VII-1980, col. N. L. H. Krauss” (USNMNH).

###### Diagnosis.

Dorsal color black with three pale yellow spots. Protibial tooth small. Apical elytral suture dentiform.

###### Description.

***Holotype*.** Female. Length 2.8 mm, width 2.2 mm; body oval rounded, convex. Dorsal color black except head pale yellow; pronotum with lateral angles and sides yellow; each elytron with three yellow spots in discal, mid-lateral and sub-apical positions (Fig. 11). Ventral surface with head, prostermum, and metaventrite brown; abdomen yellowish brown; legs, mouthparts, and antennae yellow. Head punctures small, separated by their diameter or less, each puncture as large as an eye facet; pronotal punctures larger than head punctures, separated by their diameter or less; elytral punctures as large as pronotum punctures, separated by their diameter; metaventral punctures as large as head punctures, separated by their diameter to two diameters. Elytral suture with apex dentiform. Clypeus curved. Carinae on prosternal process convergent, joined near the anterior prosternal border, larger than half prosternum. Protibia not flanged, basal tooth small. Basal abdominal ventrite without setal tuft. Abdomen with postcoxal line on basal abdominal ventrite slightly flattened along posterior ventrite margin, ventrite with sparse, long pubescence and small, dense punctures. Genitalia not examined.

Male. Unknown.

###### Variation.

Unknown.

###### Etiology.

The name refers to its similarity to other *Brachiacantha* species.

###### Type locality.

GUATEMALA, Sacatepequez: Antigua Guatemala, 14°33'N, 90°44'W.

###### Distribution.

Guatemala.

###### Discussion.

This species is very similar to *B. bistripustulata* and allies, but the pale-colored head and the yellowish ventral color of *mimica* marks the difference. The apical dentate elytral suture is shared only with *B. papiliona*.

##### Brachiacantha gorhami

Taxon classificationAnimaliaColeopteraCoccinellidae

(Weise) comb. nov.

24119BE8-686A-536E-818D-44DC8BEFDAC9

Figure 12

Coccinella pantherina Gorham, 1892: 161, tab. IX, fig. 9 (non Coccinella pantherina Linnaeus, 1758: 368) (non Coccinella pantherina DeGeer, 1773).
Coccinella gorhami Weise, 1904: 357.

###### Material examined.

El Salvador • 1♀; Montecristo, 23 km N of Metapan; 2300 m; 10 May 1971; H. F. Howden leg. (USNMNH).

###### Diagnosis.

Dorsal color pale yellow except head with brown clypeus and black above the eyes; pronotum with a black macula on the posterior margin and two large convergent subtriangular spots at center; scutellar shield black; each elytron with five black marks: a vitta on the elytral suture extending laterally in the second half of the elytron, an oval spot at the humeral callus, an elongated spot parallel to the sutural margin in the basal half of the elytron, an oval spot at middle and a small spot on the lateral margin in the apical half of the elytron (Fig. 12). Ventral surface brown. Protibial tooth small.

###### Variation.

Unknown.

###### Distribution.

El Salvador and Guatemala. New record to El Salvador.

###### Discussion.

The material was identified using the descriptions and tables of Gorham. The species was described by Gorham as *Coccinella pantherina*, but the name was preoccupied by a junior synonym of *Adalia bipunctata* (Linnaeus, 1758) and a junior synonym of *Adalia decempunctata* (DeGeer, 1773). Weise (1903) noticed the homonymy and created the name *Coccinella gorhami* for the species.

Although Gorham placed the species as a member of the genus *Coccinella*, he compared it with the species *B. lepida*, thus indicating the small size of the species. In the last century, several species originally described as *Coccinella* have been revised and assigned to other genera such as *Mulsantina* and *Cycloneda*, but there are still several species which need revision. The specimen examined corresponded to the description and figures of Gorham (1892), but the presence of eye emarginated by the eye canthus, the lack of large abdominal primary pores between fourth and fifth ventrites and the presence of a small tibial tooth identified the species as a member of *Brachiacantha*. The dorsal coloration is unique among *Brachiacantha* species but is similar to those of other genera like *Dilatitibialis*, *Serratitibia*, and *Cyrea*. A revision of the original material may be needed to confirm the taxonomic changes.

**Figures 21–24.**
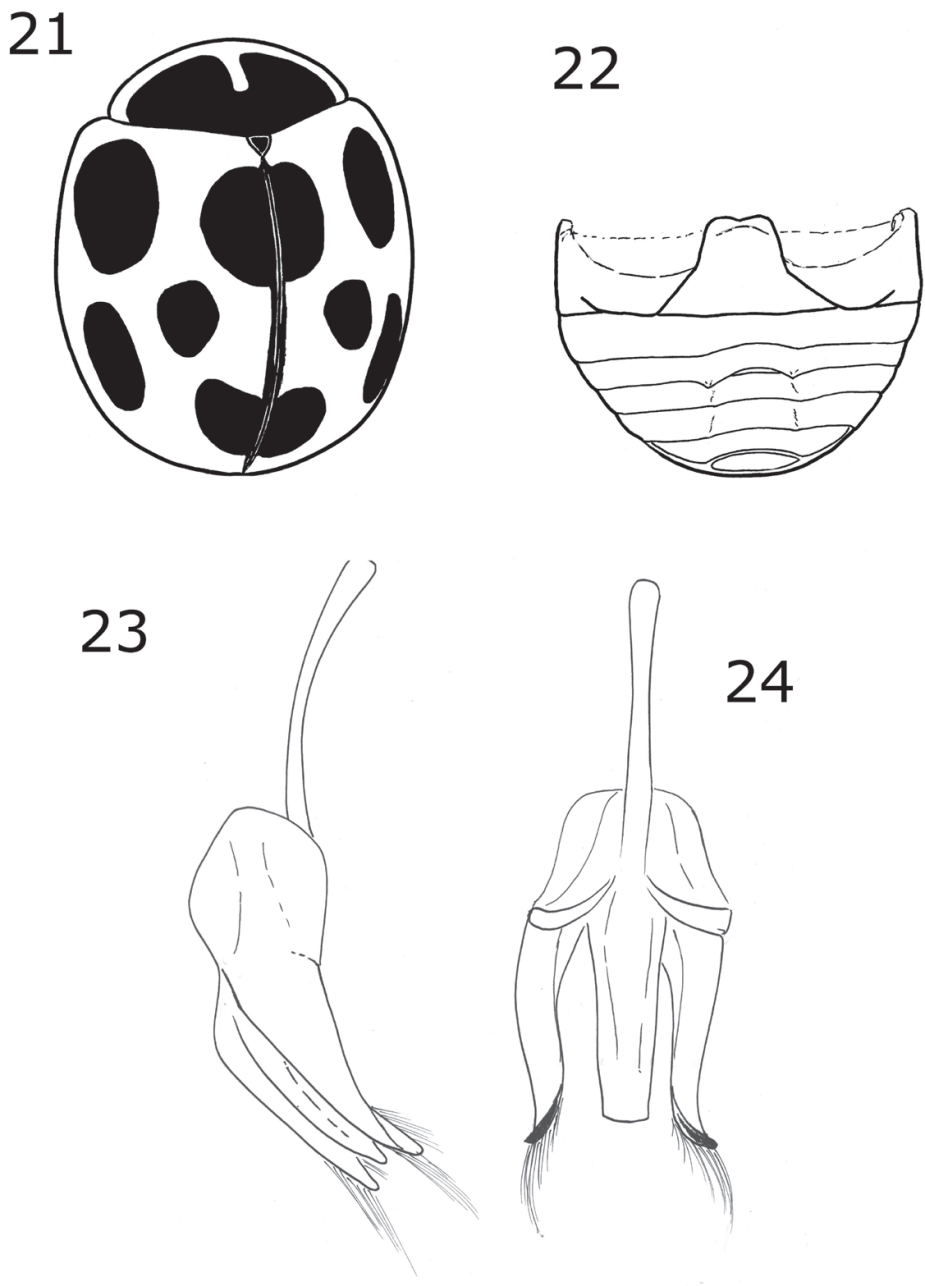
*Brachiacantha nubes* sp. nov., male **21** Dorsal view **22** Abdomen **23** Tegmen, lateral view **24** Tegmen, concave side view.

**Figures 25–28.**
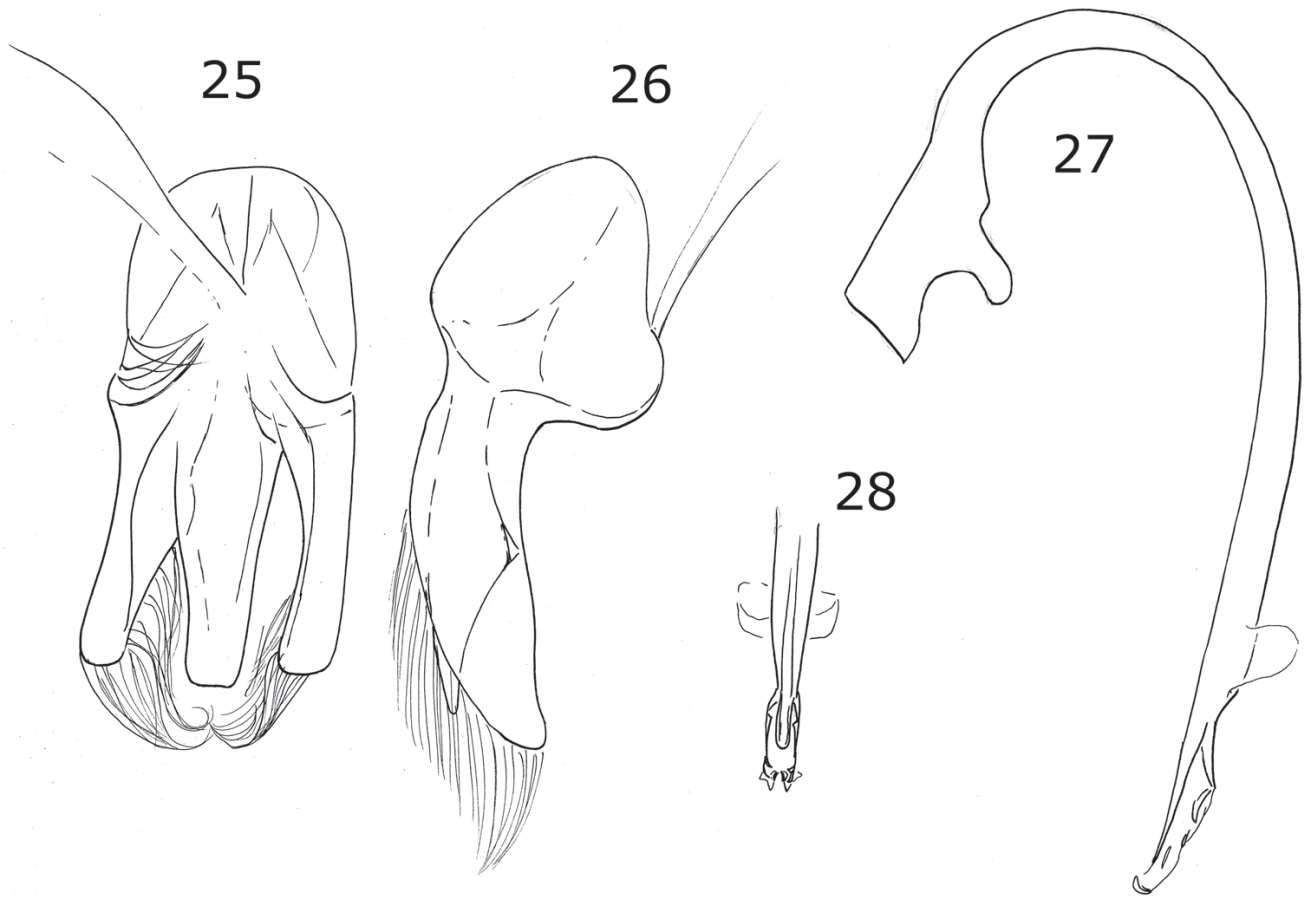
*Brachiacantha fenestrata*, male genitalia **25** Tegmen **26** Tegmen lateral view **27** Penis **28** Penis apex.

**Figures 29–32.**
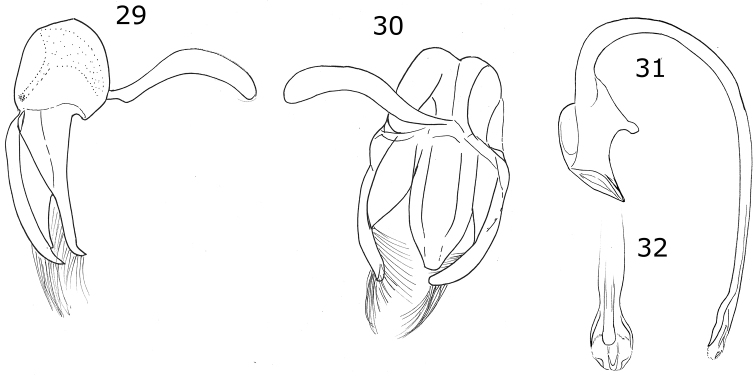
*Brachiacantha octostigma*, male genitalia **29** Tegmen, lateral view **30** Tegmen **31** Penis **32** Penis apex.

**Figures 33–36.**
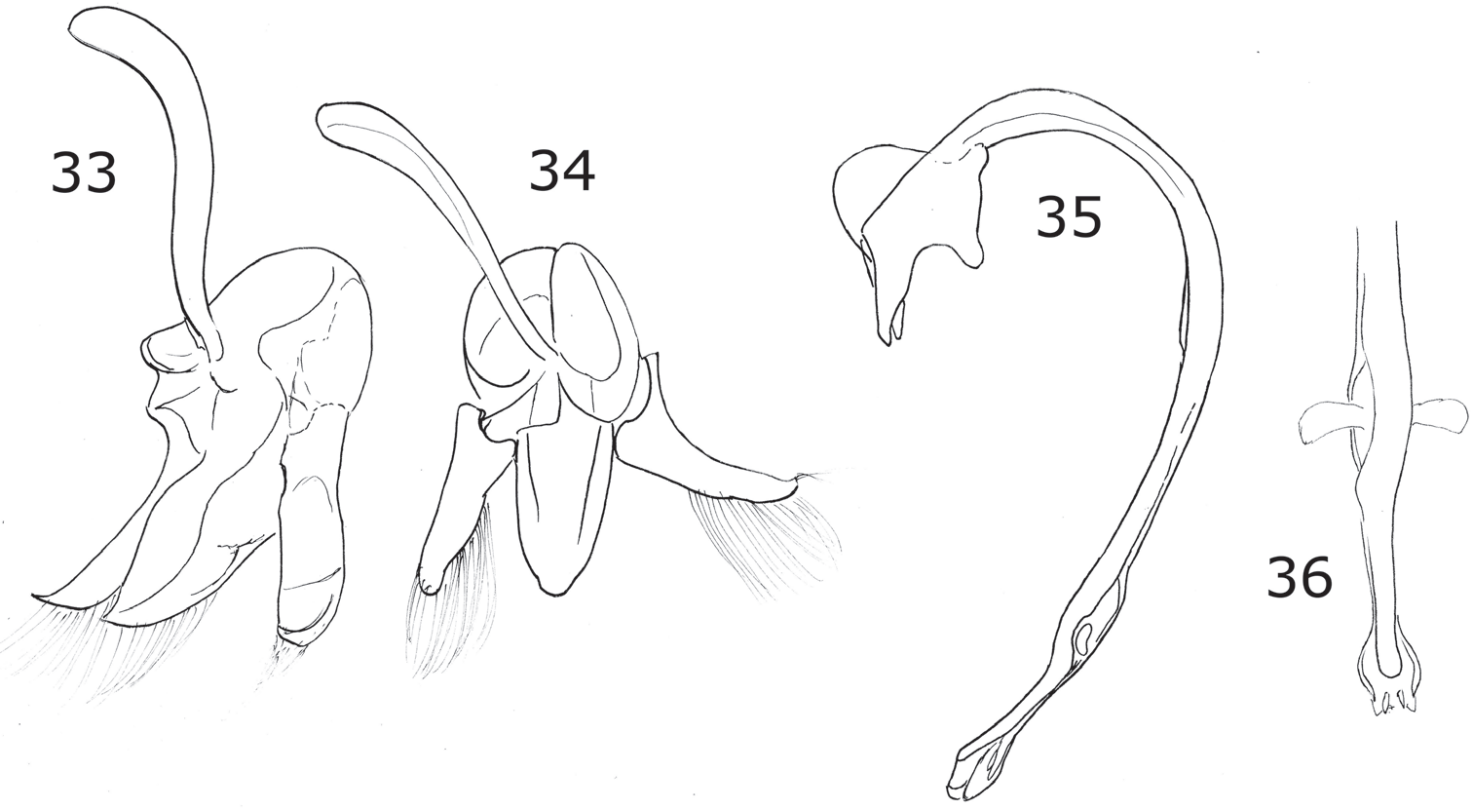
*Brachiacantha bipartita*, male genitalia **33** Tegmen, lateral view **34** Tegmen **35** Penis **36** Penis apex.

**Figures 37–40.**
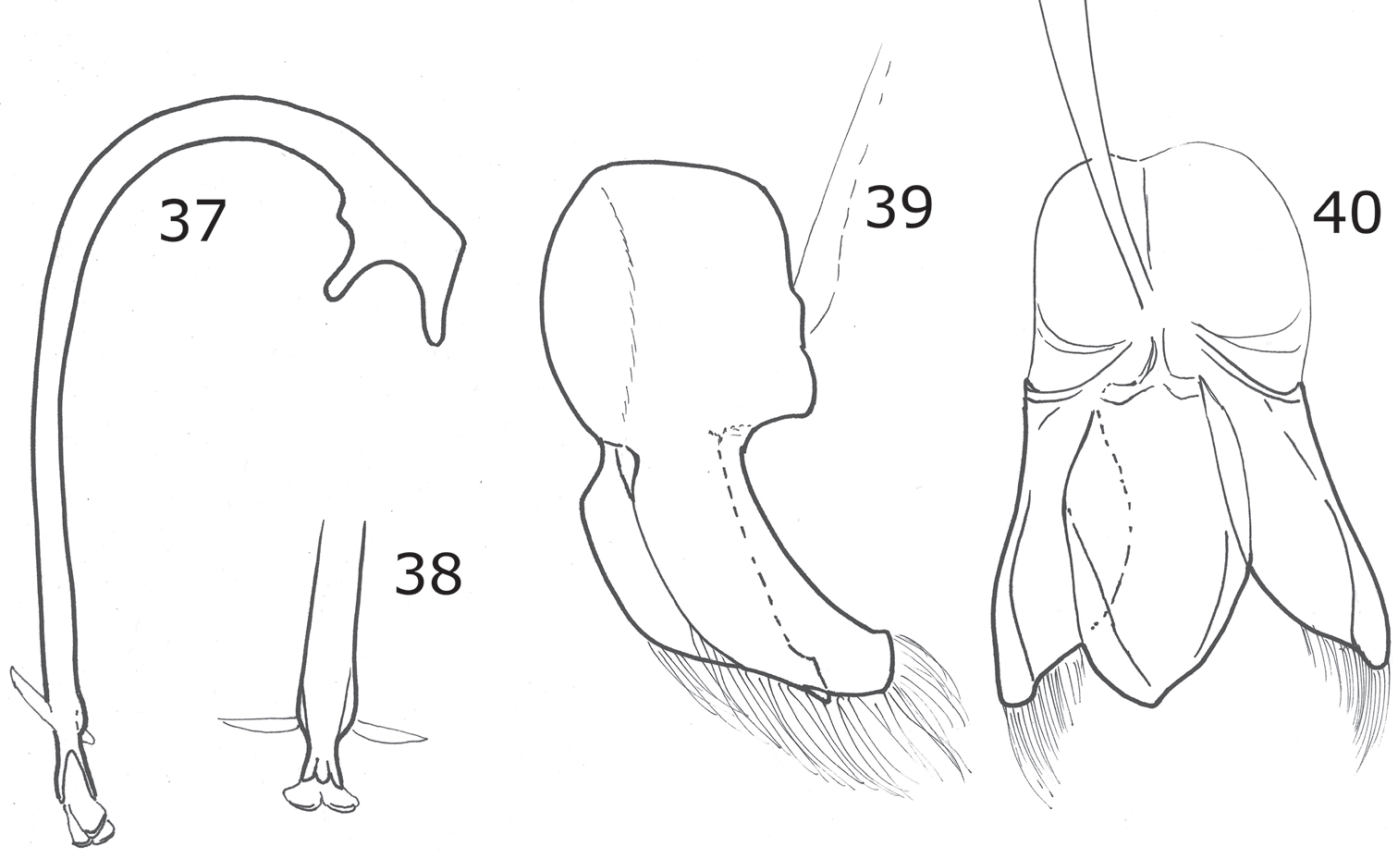
*Brachiacantha aperta*, male genitalia **37** Penis **38** Penis apex **39** Tegmen, lateral view **40** Tegmen.

**Figures 41–45.**
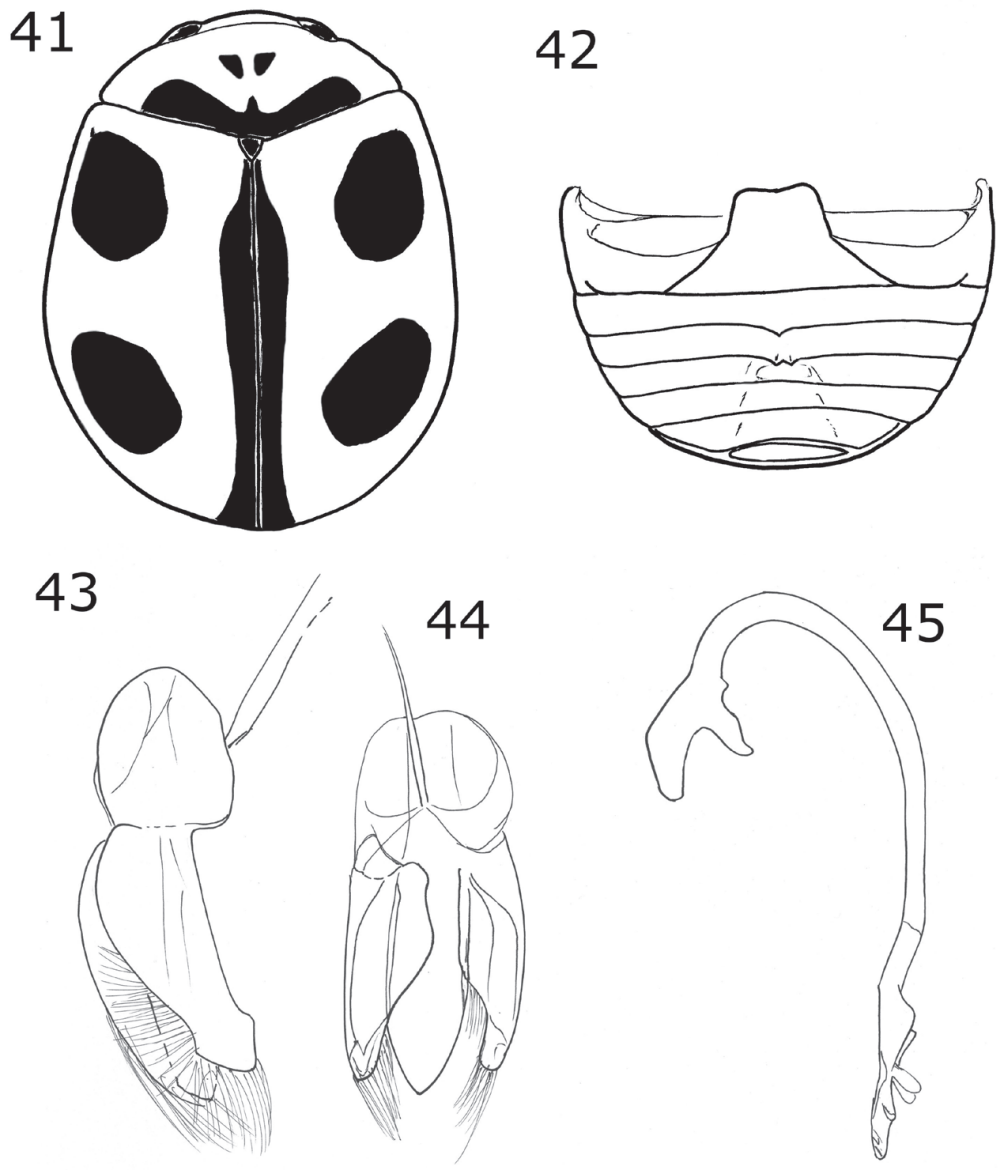
*Brachiacantha dentata* sp. nov., male **41** Dorsal view **42** Abdomen **43** Tegmen, lateral view **44** Tegmen **45** Penis.

**Figures 46–51.**
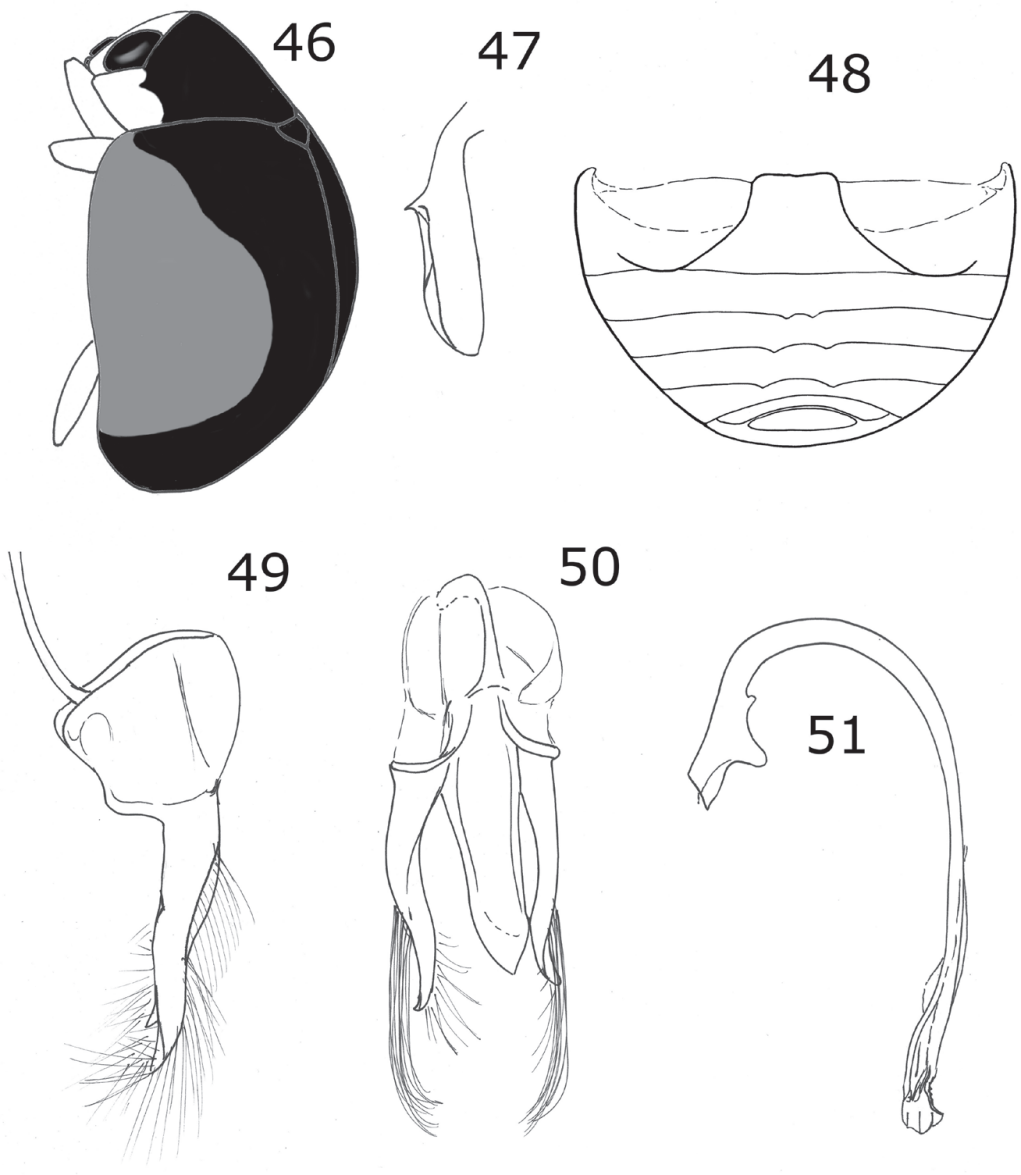
*Brachiacantha aurantiapleura* sp. nov., male **46** Lateral view **47** Protibia **48** Abdomen **49** Tegmen, lateral view **50** Tegmen **51** Penis.

**Figures 52–55.**
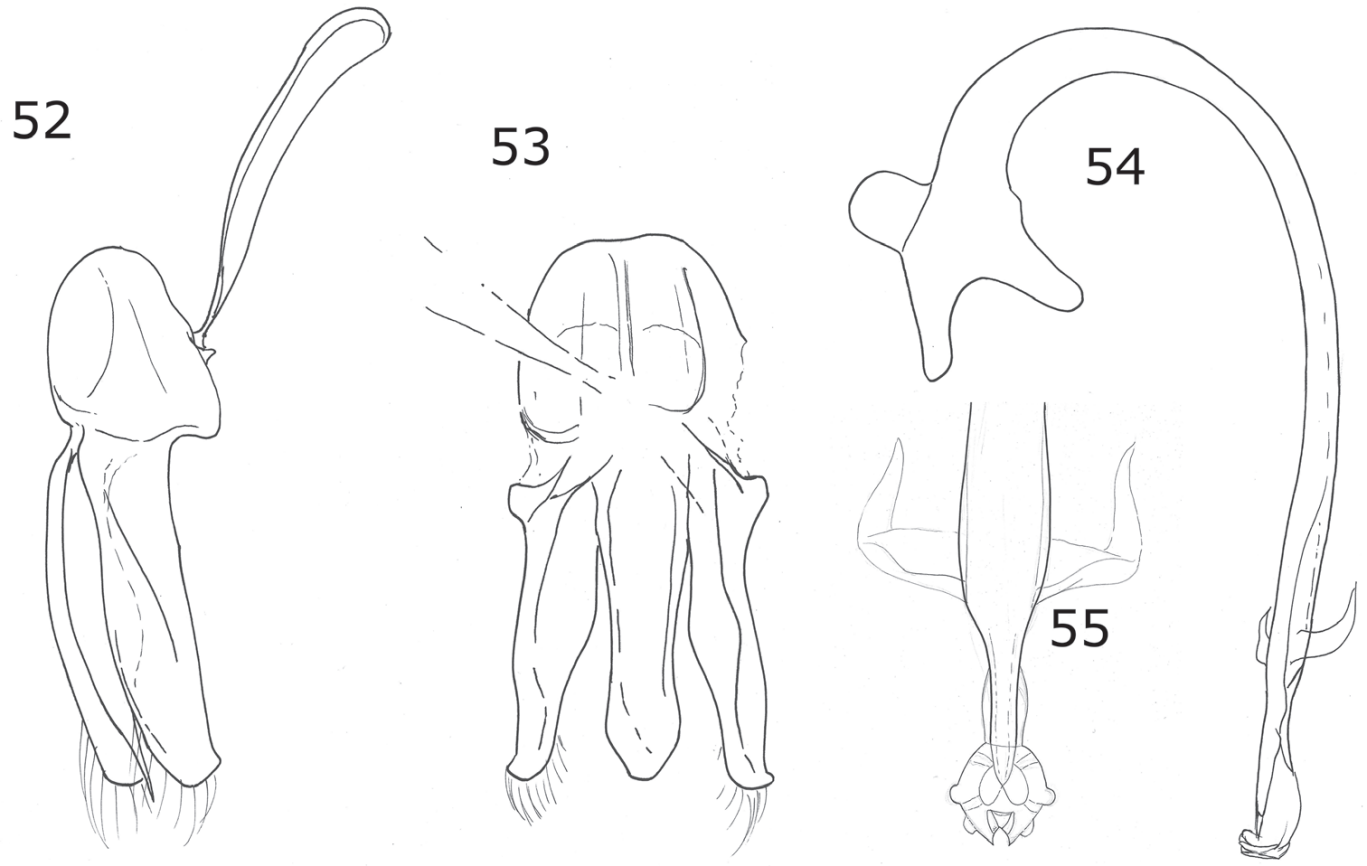
*Brachiacantha isthmena* sp. nov., male genitalia **52** Tegmen, lateral view **53** Tegmen **54** Penis **55** Penis apex.

**Figures 56–58.**
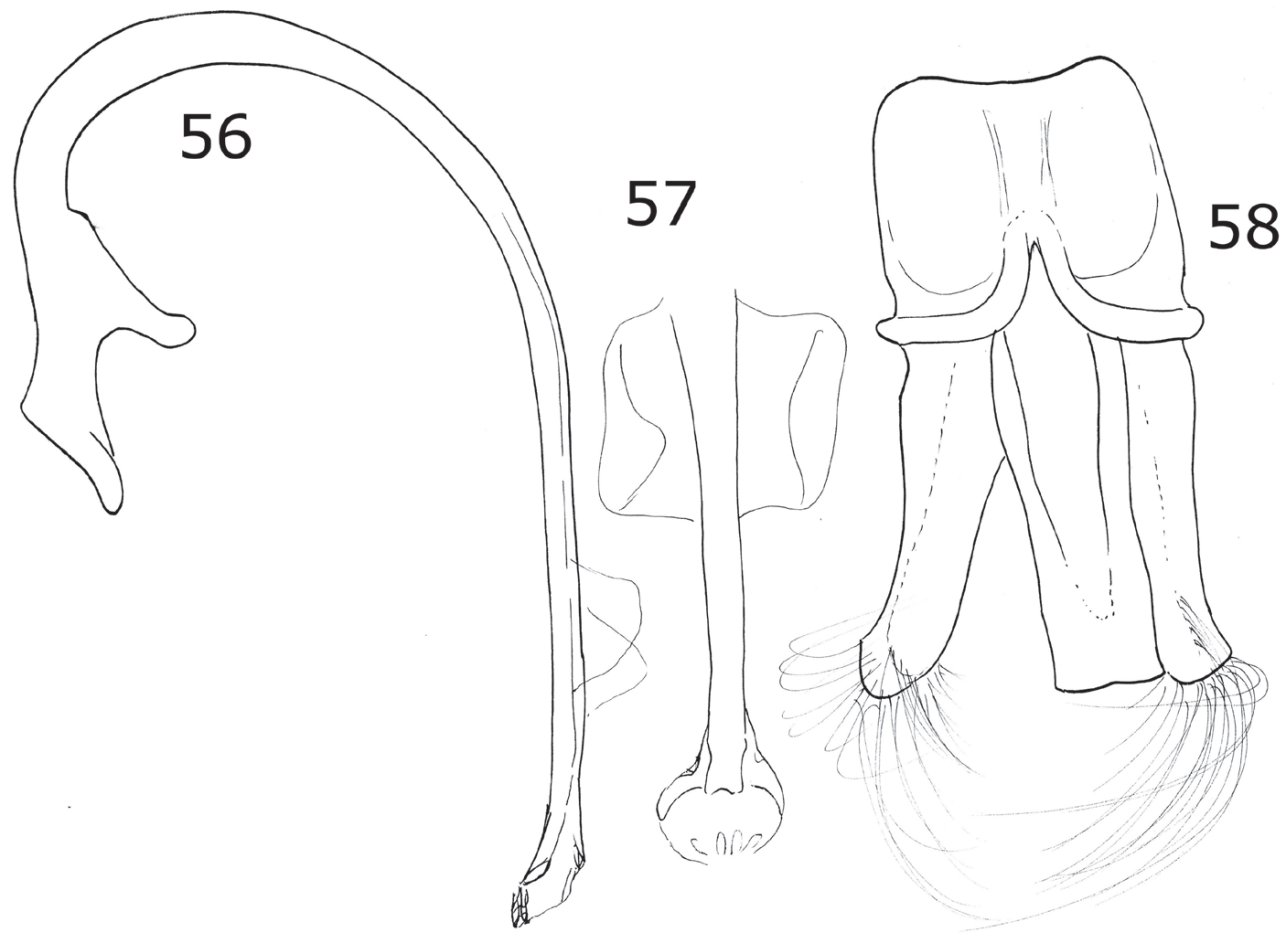
*Brachiacantha guatemalensis* comb. nov., male **56** Penis **57** Penis apex **58** Tegmen.

**Figures 59–64.**
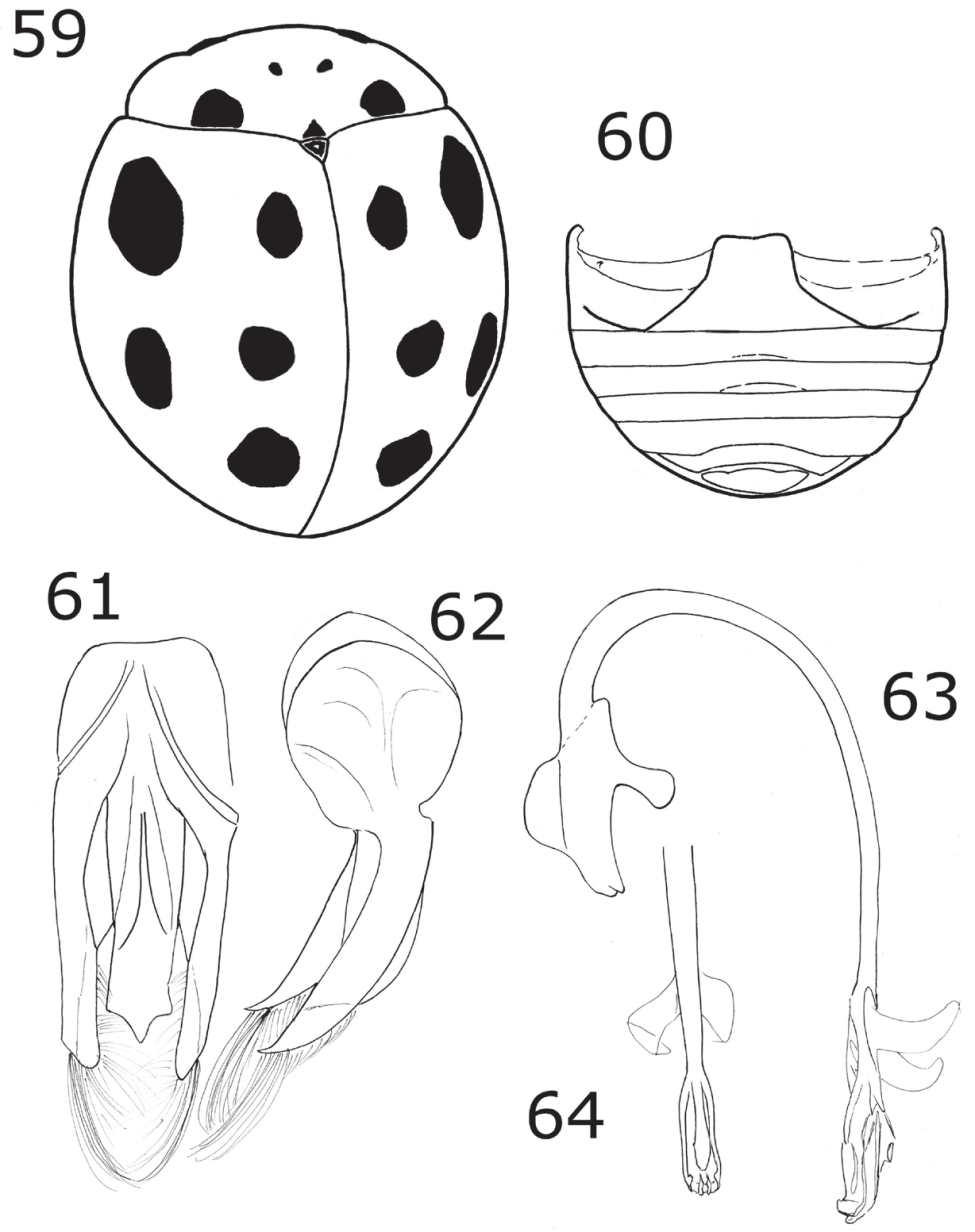
*Brachiacantha invertita* sp. nov., male **59** Dorsal view **60** Abdomen **61** Tegmen **62** Tegmen lateral view **63** Penis **64** Penis apex.

**Figures 65–68.**
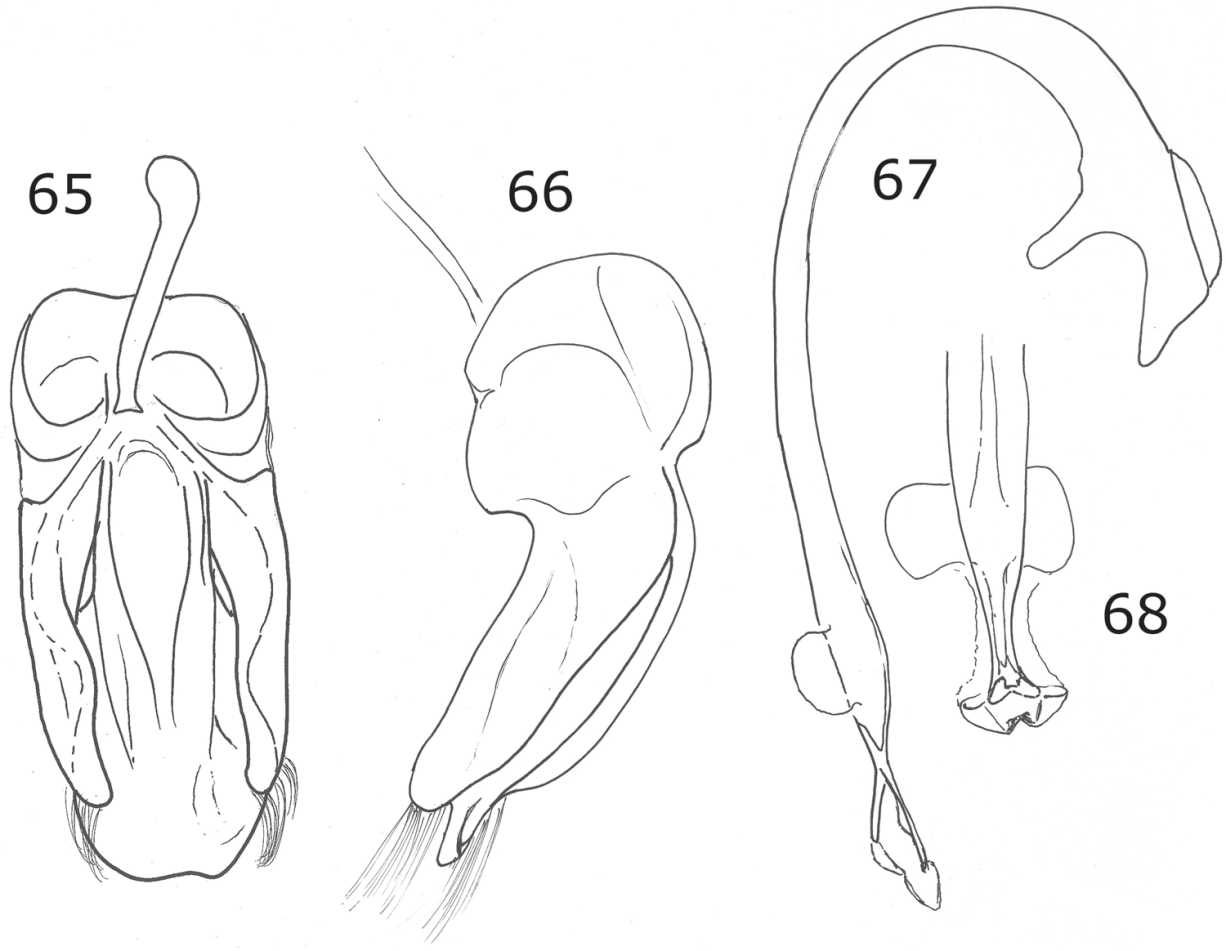
*Brachiacantha cachensis*, male genitalia **65** Tegmen **66** Tegmen lateral view **67** Penis **68** Penis apex.

**Figures 69, 70.**
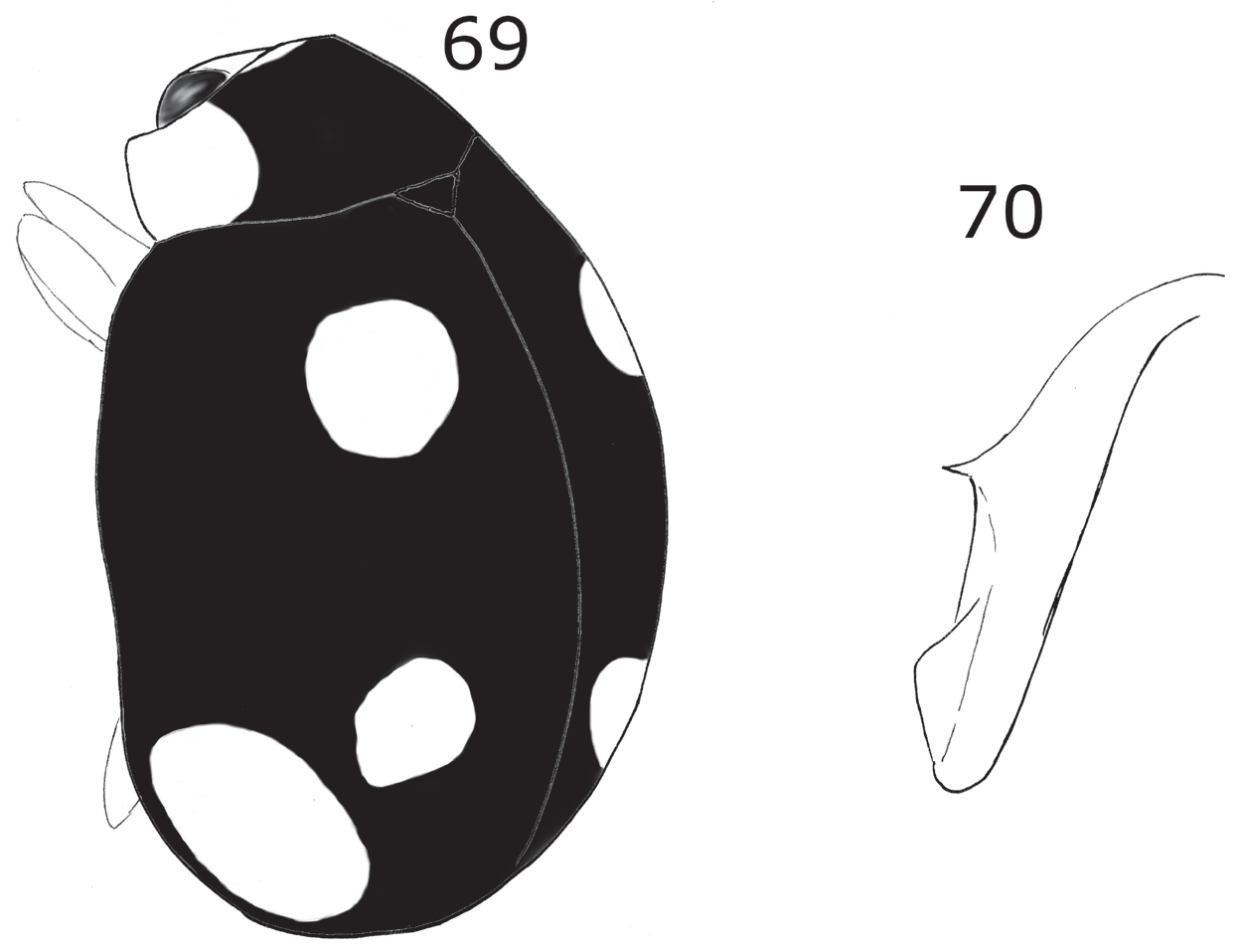
*Brachiacantha tica* sp. nov., female **69** Lateral view **70** Protibia.

## Discussion

Twenty-five species of the genus *Brachiacantha* are found in Central America, including nine new species. Some species such as *B. fenestrata*, *B. cachensis*, or *B. aperta* are easily identifiable without doubt; however, a revision of the original material is pending for the species *B. guatemalensis* (Gorham), comb. nov. and *B. gorhami* (Weise), comb. nov. Although it is not advisable to describe species based only on female specimens, the species *B. papiliona* sp. nov. and *B. tica* sp. nov. have enough external characters to ensure that they were not previously described. The species *B. mimica* sp. nov. has a very common dorsal coloration for *Brachiacantha* and *Hyperaspis* species, but the apical tooth of the elytral suture has not been described for Coccinellidae species with the exception of *B. papiliona*.

## Conclusions

Our knowledge of the Coccinellidae species in both North America and South America, has changed in the last century; however, Central America is still poorly studied. In this study several new species were discovered by reviewing collections of the United States (USNMNH, FSCA), England (OUMNH), México (CEAM, CNIN), Costa Rica (MNCR, MUCR, MZCR), and Czech Republic (NMP), but there are poorly known collections in Central American countries and the biodiversity crisis makes the need for new studies urgent to record the biological diversity of the region. A detailed study is needed of the phylogenetic relationships in the genus *Brachiacantha* to elucidate the origin of the male genitalia diversity within the genus and if the species groups are monophyletic units.

## Supplementary Material

XML Treatment for Brachiacantha barberi

XML Treatment for Brachiacantha bistripustulata

XML Treatment for Brachiacantha dentipes

XML Treatment for Brachiacantha erythrura

XML Treatment for Brachiacantha robustihamata

XML Treatment for Brachiacantha subfasciata

XML Treatment for Brachiacantha truncata

XML Treatment for Brachiacantha lepida

XML Treatment for Brachiacantha indubitabilis

XML Treatment for Brachiacantha nubes

XML Treatment for Brachiacantha fenestrata

XML Treatment for Brachiacantha octostigma

XML Treatment for Brachiacantha bipartita

XML Treatment for Brachiacantha aperta

XML Treatment for Brachiacantha dentata

XML Treatment for Brachiacantha aurantiapleura

XML Treatment for Brachiacantha isthmena

XML Treatment for Brachiacantha guatemalensis

XML Treatment for Brachiacantha invertita

XML Treatment for Brachiacantha cachensis

XML Treatment for Brachiacantha hexaspina

XML Treatment for Brachiacantha papiliona

XML Treatment for Brachiacantha tica

XML Treatment for Brachiacantha mimica

XML Treatment for Brachiacantha gorhami
